# Modern Nanocomposites and Hybrids as Electrode Materials Used in Energy Carriers

**DOI:** 10.3390/nano11020538

**Published:** 2021-02-19

**Authors:** Beata Kurc, Marita Pigłowska, Łukasz Rymaniak, Paweł Fuć

**Affiliations:** 1 Institute of Chemistry and Electrochemistry, Faculty of Chemical Technology, Poznan University of Technology, Berdychowo 4, PL-60965 Poznan, Poland; marita.piglowska@student.put.poznan.pl; 2 Institute of Combustion Engines and Powertrains, Faculty of Civil and Transport Engineering, Poznan University of Technology, Piotrowo 3, PL-60965 Poznan, Poland; lukasz.rymaniak@put.poznan.pl (Ł.R.); pawel.fuc@put.poznan.pl (P.F.)

**Keywords:** nanocomposites, hybrids, batteries, solar cells, supercapacitors

## Abstract

Over the past decades, the application of new hybrid materials in energy storage systems has seen significant development. The efforts have been made to improve electrochemical performance, cyclic stability, and cell life. To achieve this, attempts have been made to modify existing electrode materials. This was achieved by using nano-scale materials. A reduction of size enabled an obtainment of changes of conductivity, efficient energy storage and/or conversion (better kinetics), emergence of superparamagnetism, and the enhancement of optical properties, resulting in better electrochemical performance. The design of hybrid heterostructures enabled taking full advantage of each component, synergistic effect, and interaction between components, resulting in better cycle stability and conductivity. Nowadays, nanocomposite has ended up one of the foremost prevalent materials with potential applications in batteries, flexible cells, fuel cells, photovoltaic cells, and photocatalysis. The main goal of this review is to highlight a new progress of different hybrid materials, nanocomposites (also polymeric) used in lithium-ion (LIBs) and sodium-ion (NIBs) cells, solar cells, supercapacitors, and fuel cells and their electrochemical performance.

## 1. Introduction

In recent years, the biggest challenge for the electrochemical branch has been the research into new electrode materials, electrolytes, separators, or modification of the existing ones in electrochemical systems. It is related to the desire to obtain the best possible parameters during the cell’s operation, especially the specific capacity, cyclic stability, and Columbic efficiency. There are many materials that have limitations, such as limited capacity or changes in crystal structure and volume expansion during cycling. To prevent this, hybrid materials and nanocomposites are used as electrode materials to eliminate weak points of individually used systems. The term hybrid material is used in systems, such as crystalline highly ordered coordination polymers, amorphous sol-gel compounds, and materials with and without interactions between the inorganic and organic units. Between building units there are different interactions: van der Waals (50 kJ mol^−1^), H-bonding (5–65 kJ mol^−1^), coordination bonding (5–200 kJ mol^−1^), ionic (50–250 kJ mol^−1^), and covalent (350 kJ mol^−1^) [1]. There are different methods for obtaining hybrid materials.

In situ formation of inorganic materials: sol-gel process, nonhydrolytic sol-gel process, sol-gel reactions of non-silicates, combining the sol-gel approach, and organic polymers.

✓ formation of organic polymers in presence of preformed inorganic materials;
✓ formation of organic polymers in presence of preformed inorganic materials;
✓ simultaneous formation of both components; and,
✓ building block approach: inorganic and organic building blocks.
✓ To obtain polymer nanocomposites, various processes are used:
✓ mixing of dispersed particles with polymers in liquids;
✓ mixing of particles with monomers followed by polymerization;
✓ nanocomposite formation by means of molten or solid polymers; and,
✓ concomitant formation of particles and polymers.

It should be noted that there is a difference between inorganic-organic hybrids and inorganic-organic nanocomposites: term nanocomposite is used when structural units are in the range of 1–100 nm, where there are hybrid materials, and when the inorganic units are made using molecular precursors via sol-gel methods [1]. Recently, natural hybrid materials promoting the slogan of Green Chemistry are very popular, e.g., bones (three-phase composite), dentin (tissue in human tooth), nacre (mother of pearl), wood (based on cellulose), and artificial hybrid biomaterials (ancient materials) [2].

There are some requirements that electrode materials have to meet according to the type of cell. The cathode material in LIB should show high free energy of reaction with lithium (results in high voltage), incorporate a high amount of Li, exhibit high electronic conductivity, and be non-toxic as well as inert toward the electrolyte [3]. In general, anode materials dictate the energy density cycle life and power density of the cell. Among the parameters that rely on the type of electrode materials used in supercapacitors are capacitance and charge storage, f.e. we use often carbon materials because of high surface area, low cost, availability and electric conductivity. The electrochemical performance of an electrode material strongly relies on factors, like surface area, electrical conductivity, wetting of electrode, and the permeability of electrolyte solutions [4]. In NIBs, it is critical to develop electrode materials with large interstitial spaces within their crystallographic structure to host sodium ions and achieve satisfactory electrochemical performances, in the case of anode materials voltages should be low (0.0–1.0 V vs. Na^+^/Na) [5]. In solar cells: the right combination of inorganic and organic semiconductors should be chosen, nanostructures should be used to provide a large interface for the enhancement of the charge separation process, there should be a good contact between organic and inorganic components, a presence of a nano-structured network of a conducting polymer, and the symmetry in this blending layer should be achieved [6].

Various multifunctional hybrid nano-structured materials are currently being investigated to improve the energy density and power of next-generation storage devices. Alternative energy sources are renewable sources, from which energy can be obtained without being dependent on commercial suppliers and without fear that this energy will run out. In the era of increasing energy bills and greater awareness of environmental protection and climate change, alternative sources are more widely used and the demand for them is growing.

## 2. Results

Nanocomposites and hybrids for Li-ion batteries have many potential applications, which include the following:

➢ transportation: facilitate replacement of gasoline powered passenger, military, and mass transit vehicles with *Hybrid electric vehicles* (HEVs), Plug-in hybrid *electric vehicles* (PHEVs), and, ultimately, all-electric vehicles; and,
➢ utilities: safe and reliable stationary energy storage.

### 2.1. Hybrid Materials

**Figure 1:**
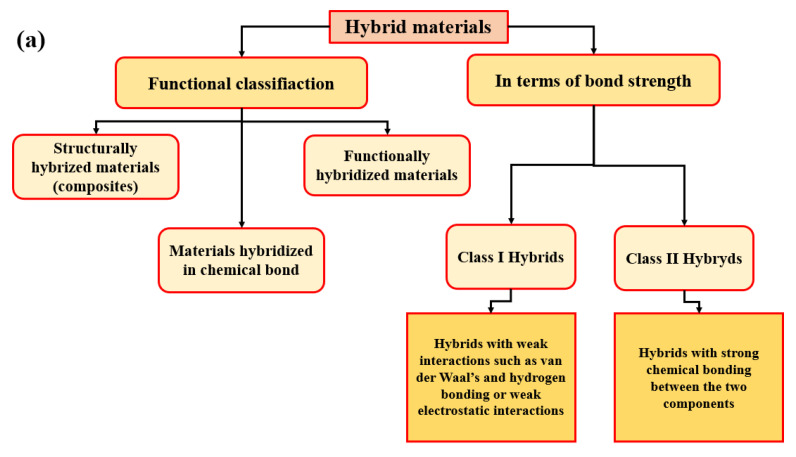

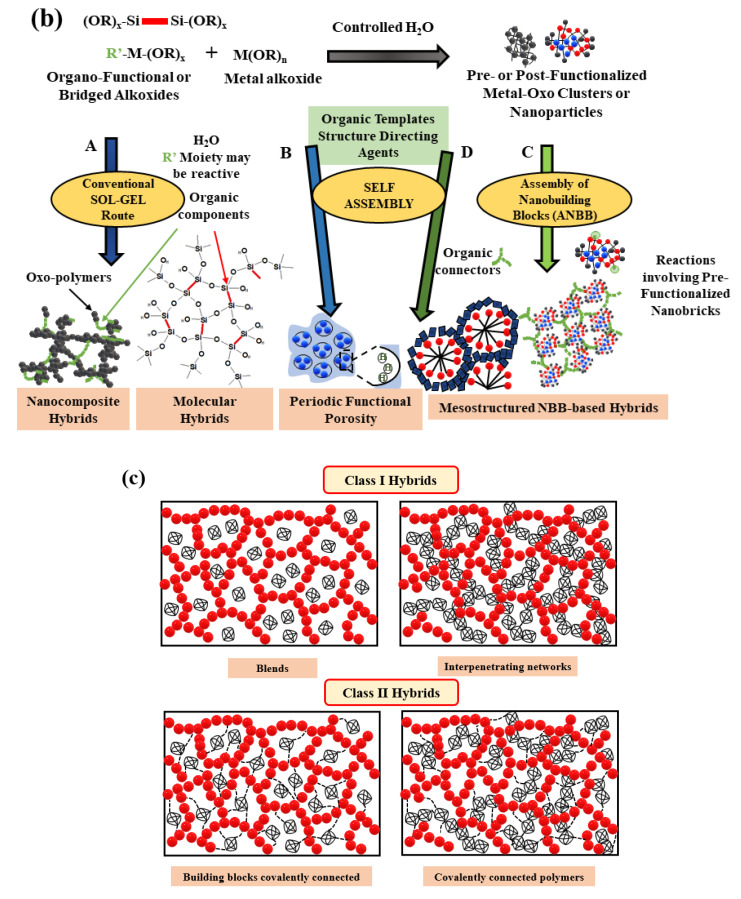
Classification of hybrids: (**a**) general classification [8]; (**b**) several general approaches for the design of sol-gel derived hybrid materials [9]; and (**c**) in terms of bond strength [10].

Hybrid materials can be defined as combinations of two (or more) materials, of materials and space and composites are connected at the nanometer or molecular level. They are assembled in such a way as to have attributes that are not offered by any one material separately [7]. Figure 1a illustrates the general classification of hybrid materials (functional classification and in terms of bond strength).

Figure 1b summarizes one key factor in the development of hybrid materials in the understanding and control of synthetic mechanisms and approaches, which allows a design of tailor-made materials with predictable properties for specific application. The use of bridged precursors of silsesquioxanes X_3_Si-R′-SiX_3_ (R′ is an organic spacer, X = Cl, Br, -OR) and route A shows the making of homogenous molecular organic-inorganic materials. Route B takes self-assembling procedures into account (versatility in creating of a whole continuous range of nanocomposites). Route C presents the assembling of well-defined nanobuilding blocks (NBB) (lower reactivity towards hydrolysis, perfectly defined structures). Route D shows the combination of self-assembly and NBB approaches (theme of synthesis with construction, covalent bonding, electrostatic interactions) [9]. The Figure 1c presents classification of hybrids in terms and of bond’s strength, which are described in Figure 1a.

In the Table 1 are placed selected hybrid materials, which are used as electrode materials in lithium-ion cell (LIB), sodium-ion cell (NIB), supercapacitors (SCs), fuel cells, and supercabatteries. 

### 2.2. Polymeric Nanocomposites 

**Figure 2:**
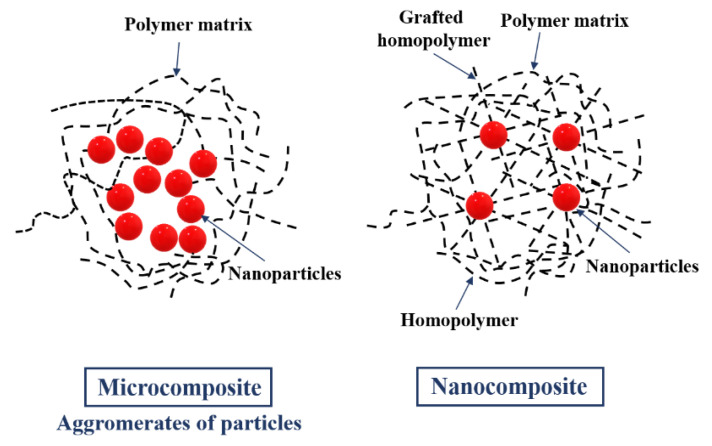
Structures of micro- and nanocomposites.

Figure 2 shows the structural differences in microcomposite and nanocomposite. It shows that the microcomposite consists of polymer matrix and nanoparticles, while the nanocomposite has an additionally grafted homopolymer and homopolymer in its structure. Generally, nanocomposites can be defined as multicomponent materials comprising multiple different (nongaseous) phase domains, in which at least one type of phase domain is a continuous phase, and in which at least one of the phases has at least one dimension of the order of nanometers [26]. In fact, the nanocomposites could be classified according to the presence of polymer (polymer-based or non-polymer based nanocomposites). In polymer-based nanocomposites, we could distinguish polymer/ceramic nanocomposite, polymer/polymer nanocomposite, polymer/layered silicate nanocomposite, biocomposites, inorganic/organic polymer nanocomposite, and inorganic/organic hybrid nanocomposite. Non-polymer based nanocomposites may be classified in metal/metal nanocomposite, ceramic/ceramic nanocomposite, and metal/ceramic nanocomposite. On the basis of reinforcement fabric material, they could be classified in metal oxide-based nanocomposite, polymer-based nanocomposite, carbon nanotube-based nanocomposite, and noble metal-based nanocomposite [27]. 

Polymeric nanocomposite (PNC) is a bi-phase material that is composed of a polymer matrix in which particles of a filler (having at least one nanometric dimension, i.e., silicas) are uniformly distributed. The polymer matrix could be made of elastomers or plastomers (thermoplasts and thermosets). They have a lot of good sites: i.e., good optical properties, size stability, reduced presence of surface defects, increased thermal stability, and flame resistance. Unfortunately, they exhibit a high price, the filler has a tendency to agglomerate, and they present problems with dispersion in polymer. 

Some of the conducting polymers are: polyaniline (PANI), polypyrrole (PPy), poly(3,4-ethylenedioxythiophene) (PEDOT), and polythiophene (PTh) used for energy storage, because these materials combine the good electric properties with the properties of conventional polymers, such as low cost, light weight, good processibility, mechanical flexibility, and thermal stability [28]. In supercapacitors, the fabrication of binary and ternary PNC with desired properties is the key in energy storage mechanism [3]. In Table 2, some PNCs systems that were applied in lithium-ion cells and supercapacitors were placed. 

### 2.3. Nanocomposites for Lithium-Ion Cells

The problem with the use of nanocomposites in lithium-ion cells is, among others, obtaining a homogeneous nanocomposite coating during scale-up, i.e., commercialization of the system. In addition, research is ongoing in developing a high-performance, low-cost process for incorporating nanomaterials into lithium-ion batteries. Moreover, it is still crucial to define how the structure can influence the properties of the electrodes.

There are ongoing efforts to develop nanocomposite materials that can significantly improve the battery performance, but further scaling and composition optimization is needed before these materials are ready for the market.

Each lithium-ion cell consists of current collectors, a current source, an anode, a separator, a cathode, and an electrolyte solution. The classical electrolyte is LiPF_6_ and Lithium is the counter electrode. The charging process involves the intercalation of lithium ions into the structure of the active anode material without changing the crystal structure, while deintercalation takes place during discharge. The classic anode active material is graphite, which has limited specific capacity (372 mAh g^−1^) and, therefore, attempts are being made to replace it with other materials. For anode materials nano-scale electrode materials could increase the storage sites of lithium ions and diffusion rate [39]. Some of them are graphene-, Si-, LiF-Fe, Li_3_AlH_6_-Al-, and VO_x_NTs-polyaniline-based nanocomposites, which are described below.

#### 2.3.1. Anode Materials 

The anode of lithium-ion batteries is usually made of carbon in the form of graphite. The energy density that can be achieved with negative electrode batteries from this material is typically from 200 to 250 Wh kg−1. They are considered it safer than lithium anode batteries. Nevertheless, it is in this last material that very high hopes are placed. This is because it has properties that are particularly desirable for anode materials in high capacity batteries. These are: high gravimetric capacity, which is 3860 mAh g−1, low density (0.59 g cm^−3^), and low electrochemical potential.

The electrode and carbon materials used in the construction of lithium batteries determine the operating parameters of the cells. Therefore, intensive work is underway for improving them, and new, active materials are still searched for, being characterized by: a good reversibility of the charge and discharge reactions, which will ensure a long cell life; high specific capacity, maintained for as many cycles as possible; mixed ion-electron conductivity; high chemical stability affecting safety (they cannot react with the electrolyte); easy to obtain; low toxicity; or, nuisance to the environment. We have selected a few examples that we think are worth presenting.

##### Graphene-Based Nanocomposites 

Carbon materials have a really few good sites: low cost, easy preparation, good conductivity, and multiple forms. Graphene is composed of a single atomic layer of graphite and it has excellent mechanical, electrical, and optical properties. Unfortunately, it has some practical limitations: low electron/lithium ion transport between sheets resulting in worse electrochemical performance of anodes. Additionally, graphene has a huge specific surface area, which, on the one side, enables a good capacity, but on the other hand it could cause in agglomeration between sheets and, thus, reduce the effective area and capacity. Because of those limitations, graphene is only examined as a hybrid or nanocomposite compound or in modified form. However, it is well known that graphene could be also used as a conductive carrier and connect the active materials because of its highly good mechanical properties, thus preventing the destruction of electrode structure [40]. The three-dimensional (3D) conductive network (formed by graphene) may improve the electron and ion movement within the electrode materials [41].

**Figure 3:**
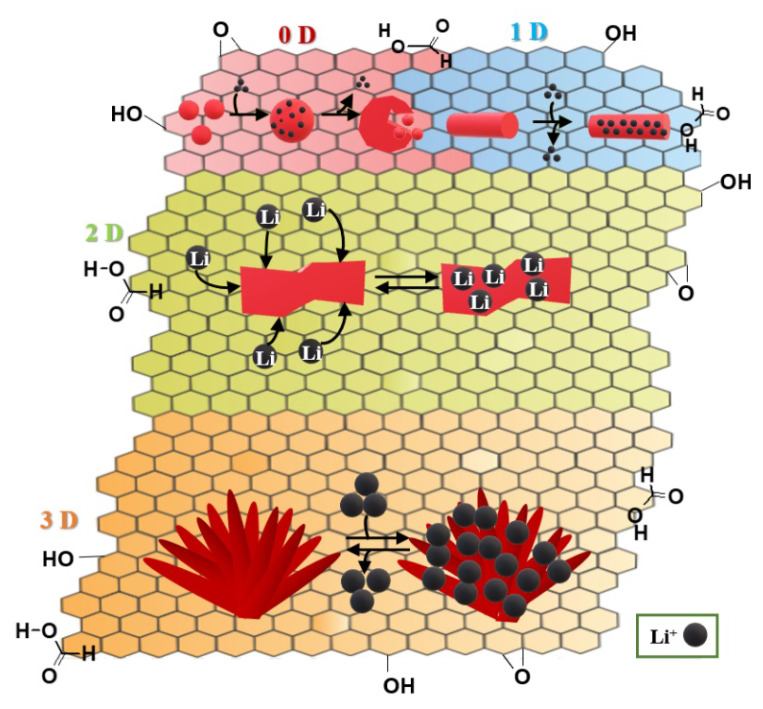
Zero-dimensional (0 D), one-dimensional (1 D), two-dimensional (2 D), three-dimensional (3 D) graphene-based anode materials in LIBs and intercalation-deintercalation mechanism, based on [42].

In Figure 3, it was shown that one-dimensional (1D), two-dimensional (2D), and 3D electrodes exhibit better electrochemical properties than zero-dimensional (0D).

We could highlight a few of graphene-based nanocomposite anodes for LIBs and mechanisms of intercalation-deintercalation processes (Table 3) [42]: graphene-supported transitional metal oxides: nickel oxide, cobalt oxide, copper oxide, iron oxide, and other transitional metal oxides; graphene–Sn/Si/Ge-based nanocomposites; graphene-supported metal sulfides: applied sulfides: MoS_2_, CoS, NiS, CuS, and FeS, SnS_2_; graphene–carbon nanotube based composites.

The authors in work [43] showed the solvothermal method to be an efficient tool for the preparation of V_x_O_y_-TiO_2_-rGO materials with uniform spherical morphology. The addition of vanadium precursor to the reaction system facilitates the aggregation of particles into large conglomerates. XRD measurements indicate that the vanadium atoms are well incorporated in the TiO_2_ crystalline structure. The work has demonstrated that V_x_O_y_-TiO_2_-rGO displays improved electrochemical stability upon the reported lithiation and delithiation, which effectively improves the long-term electrochemical performance and maintains the specific capacity well. The V_x_O_y_-TiO_2_-rGO microparticles synthesized, as described here, may be a promising candidate as an anode material for future application in LIBs.

##### Si-Based Nanocomposites

**Figure 4:**
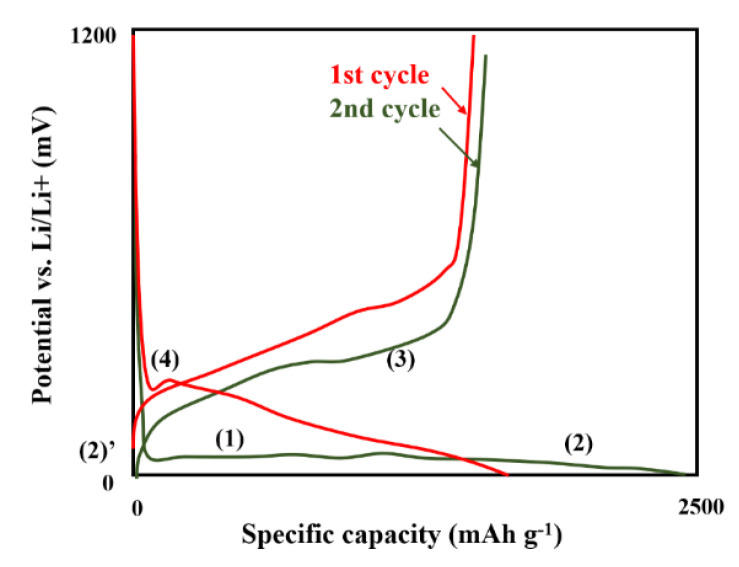
A typical charging/discharging curve for Si anode (100 nm Si electrode), based on [44] (Equations (1)–(4)).

Si is a very prospective high-performance anode for LIBs. When compared to classical graphite anode, the Si anode exhibits higher potential vs. Li/Li^+^ (0.3 V), theoretical capacity (3578 mAh g^−1^). Unfortunately, the main limitation is volume expansion during cycling. Additionally, important challenges remain desirable rate and cycling performance. The typical cycle life is in range of 200 to 300 (while for graphite > 1000). The ion storage mechanism for silicon is alloying/de-alloying (for graphite inertion/extraction). During the charge–discharge process, four plateous (Figure 4) could be observed, which arise while following reactions [44], Equations (1)–(4):

(1) $$Si\;\left(crystalline\right)+xLi\to \left(1-\frac{x}{y}\right)Si+\frac{x}{y}Li_{y}Si(amorphous)$$

(2) $$4Li_{y}Si(amorphous)+\left(15-4y\right)Li↔Li_{15}Si_{4}(crystalline)\;at\;E<60\;\mathrm{mV}\;Li_{15}Si_{4}\left(crystalline\right)↔4Li_{y}Si\left(amorphous\right)+\left(15-4y\right)Li\;at\;E<60\;\mathrm{mV}$$

(3) $$Li_{15}Si_{4}\;\left(amorphous\right)\to 4Si\left(amorphous\right)+15Li$$

(4) $$Si\left(amorphous\right)+xLi↔\left(1-\frac{x}{y}\right)Si\left(amorphous\right)+\frac{x}{y}Li_{y}Si\left(amorphous\right)$$

**Figure 5:**
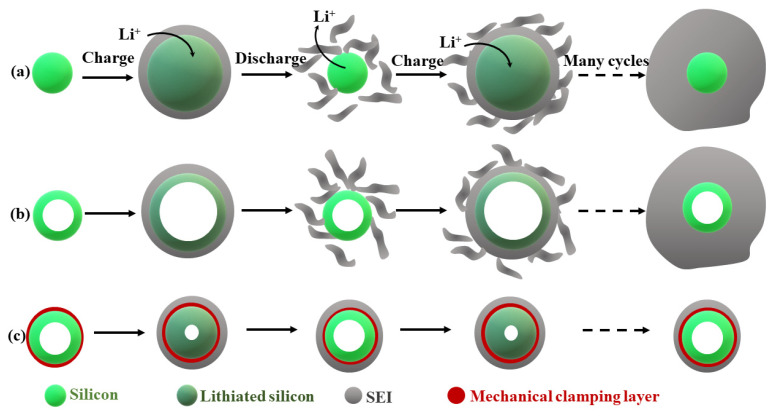
Si-based nanocomposites for anodes in LIBs and schematic of SEI formation on silicon surfaces: (**a**) solid silicon nanowire; (**b**) the silicon nanotube without a mechanical constraining layer; and, (**c**) designing a mechanical constraining layer on the hollow silicon nanotubes can prevent silicon from expanding outside toward the electrolyte during lithiation, based on [45].

Figure 5 presents silicon nanotube sorrounded by the Li^+^ permeable silicon oxide shell layer.

The oxide layer prevents the inner part of nanotube from electrolyte and provides a stable SEI layer, even after volume change od this material upon cycling, which increases the life cycle and performance up to 6000 cycles of LIBs [45].

In work [46] Munao et al. proposed Si- Carboxy-Methyl-Cellulos (CMC) nanocomposite as anode material for LIBs that were prepared by a combination of two techniques: Laser Assisted Chemical Vapor Pyrolysis and Electrospray Deposition. As performed anode exhibited a high specific capacity up to 1200 mAh g^−1^ (at C/20) and a good rate capability. Moreover, electrodes contained abundant, non-toxic, and low-cost materials with good reproducibility. Limation still remains minor morphological changes during cycling. In the article [47], a stable high-capacity and high-rate silicon-carbon (SF@G) upon 2D covalent encapsulation process was synthesized. SF@G exhibits a volumetric capacity of 2350 mAh cm^−3^ (at 0.8 A g^−1^)—four times higher than for commercial graphite anodes (550 mAh cm^−3^). The covalent bond creates an efficient contact between the Si and electrically conductive media, which enables fast electron and ion transport from the Si and back.

##### Li_3_AlH_6_-Al-Based Nanocomposites

**Figure 6:**
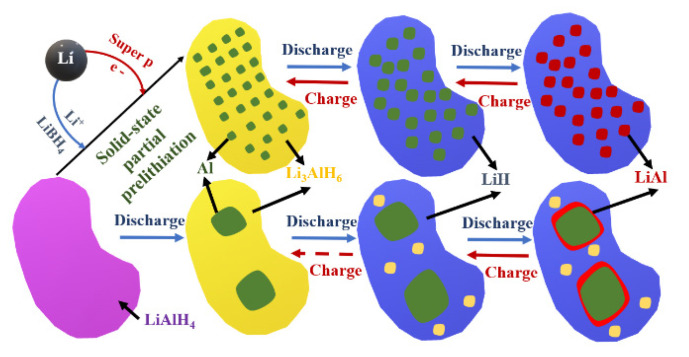
Schematic illustration of the structural evolution of the solid state partial prelithiation (SSPP) LiAlH anode and LiAlH_4_ anode during cycling in LIBs, based on [48].

Figure 6 presents the differences between solid state partial prelithiation (SSPP) Li-AlH anode and LiAlH_4_ anode upon cycling.

The partial prelithiation facilitates the fast electron and lithium ions movement through carbon and P6_3_mc LiBH_4_ and leads to a short-circuited intercalation of LiAlH_4_. During discharge, the amorphous Li_3_AlH_6_ is fully lithiated to LiH and aluminum (with Al nanograins). During charge process, Li is first fully delithiated to Al. The big adventage is that during cycling the morphological structure is well maintaned, thus resulting in cyclig stability of electrode [48].

In work [48], the authors synthesized Li_3_AlH_6_-Al nanocomposite and obtained a high specific capacity (2266 mAh g^−1^), Coulombic efficiency (88%), cycling stability (71% retention in the 100th cycle), and rate capability (1429 mAh g^−1^ at 1 A g^−1^) as a anode in LIBs. It was also shown that, during the intercalation process, reactions given by Equations (5) and (6) could be applied:

(5) $$\frac{2}{3}Li_{3}AlH_{6}+\frac{1}{3}Al+2e^{-}+2Li^{+}↔4LiH+Al$$

(6) $$4LiH+Al+e^{-}+Li^{+}↔4LiH+AlLi$$

##### Cobalt-Based Mesoporous Nanocomposites

It was shown that mesoporous lithium intercalation materials with regular porosity promote facile and fast diffusion of lithium ions, which lets achieve higher stability and rate capabilities than bulk electrodes. Nanocasting cobalt-based mesoporous electrode materials have been recently well known, i.e., nitrides, phosphides, and sulfides [49]. After some improvements, those materials could serve as promising future anodes. In work [50], the authors presented electrochemical performance for Co_3_O_4_-KIT-6-(40, 80, 100, and 130) anodes and obtained the reversible specific capacities (at 50 mA g^−1^) 943–1141 mAh g^−1^ during cycling. Additionally, m-CuCo_2_O_4_-(40, 130) were examined and those materials showed cyclic capacity (at 60 mA g^−1^) 829–1080 mAh g^−1^ and Coulombic efficiency of 59–69% [51]. In work [52], the authors proposed HOM-ZnCo_2_O_4_ and NPS-ZnCo_2_O_4_ compounds, which, as anodes, could achieve the cycling capacities of 1286–1623 mAh g^−1^ (at 2 mA g^−1^). M1-Co_3_O_4_ and M8-Co_3_O_4_ materials reached the capacities upon cycling (at 890 mA g^−1^) in a range of 790–1190 mAh g^−1^ and Coulombic efficiency of 75% [53]. Generally, it was shown that a highly ordered mesoporous structure could increase the active sides, thus resulting in better ion transport in the electrolyte/electrode interface.

##### SnO_2_-Based Nanocomposites

SnO_2_ is an important transition metal oxide that has a multifunctional electrochemical application (lithium-ion cells or sodium-ion cells). The material has attracted attention as a potential next generation anode owing to its high theoretical capacity of 1494 mAh g^−1^ for Li ions storage [54]. Unfortunately, the undesirable electrical conductivities and huge volume variations during cycling processes with Li^+^ intercalation and de-intercalation could lead to capacity fading and poor cycling stability. The intercalation mechanism could be described using Equations (7) and (8).

(7) $$SnO_{2}+4Li^{+}+4e^{-}↔2Li_{2}O+Sn$$

(8) $$Sn+xLi↔Li_{x}Sn\;(0<x<4.4)$$

Different nanocomposites are examined to solve the problems using a poor SnO_2_. In work [55], the authors showed SnO_2_/nanocomposite@TiO_2_ used as an anode in LIBs. The specific capacity after the second cycle was high and equal to 1224 mAh g^−1^ specific capacity at 0.1 A g^−1^ and capacity retention was 72.2%. The nanocomposites show much improved cycling stability and rate capability compared with the bare SnO_2_ and SnO_2_/NC electrodes. SnO_2_@C/multi-wall carbon nanotubes (MWCNTs)-lithium fluoride composite was also examined [56], and also exhibited after the second cycle a specific capacity of 700 mAh g^−1^ at 0.05 A g^−1^ and Coulombic efficiency of 70.1 up to 100%. Additionally, it was shown that MWCNTs improve the conductivity and inhibit the volume expansion. The authors in manuscript [57] showed the electrochemical performance of SnO_2_/SnS@N–C composite, which achieved a specific capacity of 1050 mAh g^−1^ at 0.1 A g^−1^ and a low capacity retention of 52.1%. Because the obtained material has a synergistic enhanced effect at heterointerfaces, which boost the charge transfer, thus promoting electrical conductivity.

##### Lignocellulosic Biomass-Based Nanocomposites

Lignocellulosic biomass, most abundantly available and green raw material, is used here to obtain a specific carbon material. Lignocellulose sources have been exploited as not only porous carbon materials and binders, but also as separators and electrolyte reservoirs [58]. It is essential to obtain a high specific surface area, rate capability, high capacity, diffusion rate in order to achieve a high power density, and it is why we have to carefully plan the preparation of carbon material process.

In work [59], the authors presented an electrochemical performance of SnO_2_/C nanocomposite/hydroxyethyl cellulose (HEC) as an anode material achieving the specific capacity values of 1074 mAh g^−1^ at 0.1 A g^−1^ and 459 mAh g^−1^ at 12.8 A g^−1^, Coulombic efficiency of 98%, and cyclic retention of capacity of 88.4% after 400 cycles at 1 A g^−1^. The following full battery testing at the voltage of 4.3 V also demonstrates its practicality. Additionally, a very interesting nanocomposite was synthesized by the authors in manuscript [60]. Binder-free SiO_x_/C composite/Kraft lignin achieved ~900 mAh g^−1^ at 100 mA g^−1^, Coulombic efficiency of 100% and capacity retention of ~100% after 250 cycles at 200 mA g^−1^). There were two aims achieved in the paper: the use of lignin as a renewable precursor for fabricating high performance Si electrodes for LIBs and pyrolized lignin with PEO as a backbone that forms a binder free matrix with brilliant electronic conductivity, ionic conductivity, and adhesion, thus preventing the use of the conventional binders.

##### Polymer-Based Nanocomposites

Microstructural electrodes are most often complex systems, e.g., composite materials, multiphase systems built using the layer-by-layer technique, and systems built with the use of supramolecular chemistry techniques. Such structures use both traditional redox systems and nanostructures that are capable of electron transferring, such as fullerenes, nanotubes, graphene, metal nanoparticles, nanoparticles of metal compounds with other elements, such as CdS (Cadmium sulfide), CdSe (Cadmium selenide), MnO_2_ (Manganese (IV) oxide), and Fe_3_O_4_ (Iron (II,III) oxide) [60]. Carbon nanoparticles, such as fullerenes, nanotubes, and graphene, may be used in their basic form, but they can also be functionalized by introducing various types of functional groups into their structure [61]. This significantly broadens the possibilities of using these nanostructures. In addition, conductive polymers are used to build microstructural electrodes. Dies are another important group of materials. The most commonly used matrices contain in their structure pores or channels of a certain size, being regularly distributed throughout the volume of the matrix. Examples of such materials are porous carbon and polycarbonate matrices. Porous carbon is a conductive material and will, therefore, suffice in putting, in its pores, a suitable material, e.g., a redox system, to obtain a ready electrode with a specific distribution of active centers on its surface. The polycarbonate matrix is non-conductive, but the so-called z-conductive layers are obtained after filling the channels that occur in its structure with a conductive material. Conductive material has various applications, including that used as an electrode material. Its basic parameters are electrical conductivity, pore diameter, and their density. If the pores are filled with a material capable of redox reaction, we will obtain an electrode with redox centers that will be able to catalyze selected processes. The number of immobilized redox centers will depend on the density of pore occurrence, their diameter, and the degree of filling them by redox systems. If we assume a constant level of the pore filling, then the properties of the electrode will be largely determined by the symmetry of the porous carbon. Another type of matrix are highly symmetrical matrices that are made of aluminum oxide or titanium oxide. These matrices consist of hexagonal columns with a channel running inside them. However, these materials are rarely used as electrode material due to the low conductivity of alumina and titanium oxide. The first step is to create a porous Al_2_O_3_ (Aluminium oxide) layer on the surface of the aluminum foil. This process is usually carried out by electrochemical oxidation. By controlling the parameters of oxidation processes, it is possible to obtain different diameters of the channels [62]. The next step is to fill the tubules with a suitable substance, e.g., a conductive polymer. Subsequently, a metallic contact is placed, i.e., the base of the electrode, e.g., gold is sputtered. The final stage is dissolving the matrix; we obtain a gold electrode that is covered with conductive polymer nanowires. Another type of matrix is the “honeycomb” layer produced on the electrode surface. An example of such an electrode is made of a composite of poly (3,4-ethylene-1,4-dioxythiophene and polystyrene sulfonic acid lithium salt (PEDOT/PSSLi) [63]. The next step is electrochemical polymerization and the formation of a PEDOT layer on the electrode covered with spheres. The thickness of the polymer layer should be comparable to the diameter of the spheres. The next stage is dissolving the polystyrene spheres. The electrode is obtained with a modified PEDOT layer with a honeycomb structure. The pores in this matrix can be filled with a material catalyzing the selected oxidation process, while the conductive PEDOT matrix provides the possibility of regeneration of the catalyst by electrode oxidation. Honeycomb systems can be made of other materials, e.g., carbon [64], diamond [65,66], metals (e.g., platinum and palladium) [67], and many others. Transition metal oxide-based on carbon-polymer anodes are used as anodes with polymer nanocomposites. An example is the coaxial MWCNTS-MnO_2_-PPy composite, which was synthesized through an in situ polymerization method [68]. The work [69] shows the synergistic effect of the MWCNT matrix and highly conductive properties of PPy coating layer. The reversible specific capacity for the composite retains 820 mAh g^−1^ after 120 cycles at a current density of 100 mA g^−1^, which is high and decides the cyclic stability of an electrochemical system. Moreover, the Sn/Si/carbon-polymer-based anodes are exposed in researches [70,71,72]. The lithium ion intercalation process takes place through an alloying reaction mechanism. Sn and Si anodes exhibit the high specific capacities of 990 mAh g^−1^ and 4200 mAh g^−1^, respectively, separately. Within Sn/carbon-polymer-based negative electrodes, we can distinguish, inter alia, the ternary ethylene glycol (EG)/SnO_2_/PANi composite by using a hydrothermal method and in situ oxidative polymerization. In the anode system, it reaches an initial current efficiency of 78% and maintains a specific capacity of 408 mAh g^−1^ after 100 cycles at 100 mA g^−1^ [73]. Within nanocomposites with silicon, the 3-D Si/PPy/CNT system can be distinguished. The system achieves a specific capacity of 1600 mAh g-1 and Coulombic efficiency of 99.9% after 1000 cycles [74].

#### 2.3.2. Cathode Materials

Lithium cobalt oxide (LiCoO_2_) is a popular material for the cathodes in the cells. Lithium-ion batteries are also available with a cathode that is made of lithium iron phosphate oxide (LiFePO_4_), lithium manganese oxide (LiMn_2_O_4_) and materials: NMC, based on lithium, nickel, manganese and cobalt (LiNiMnCoO_2_) and NCA, which in its composition, apart from lithium, nickel, and cobalt, it also includes aluminum (LiNiCoAlO_2_). 

The cathode material should:

❖ contain an ion easily undergoing redox reaction, e.g., a transition metal ion;
❖ have a high redox potential of the intercalated compound with respect to lithium. To achieve high voltage, the transition metal should have a high degree of oxidation;
❖ capable of a high speed and reversible lithium intercalation/deintercalation process to ensure long cell life;
❖ be able to reversibly incorporate a large amount of lithium (at least one atom per metal atom) into available places in the material structure to maximize the cell capacity;
❖ it is characterized by high electronic and ionic conductivity, which allows achieving minimal polarization losses during the processes of charging and discharging the battery and achieving good efficiency of the cell;
❖ be chemically stable. The electrode compound should not decompose under the cell’s operating conditions or react with the electrolyte; and,
❖ in addition, the cathode material should not be expensive, difficult to synthesize, toxic, and harmful to the environment.

LiCoO_2_ has the advantage of high ion and electronic conductivity, the disadvantage is the toxicity of the material and its significant cost. Moreover, only 50% of the theoretical capacity of this material is useful in practice, because of its chemical instability under deep charging. LiMn_2_O_4_ is also characterized by high ion and electronic conductivity and a high redox reaction rate. It is inexpensive, environmentally friendly, and safe. Unfortunately, in its case significant losses of capacity are observed at elevated temperatures. LiFePO_4_ is inexpensive and safe for both the user and the environment. However, it is characterized by a very low electron and ion conductivity. Improving these properties is achieved by reducing the size of the particles and then coating them with carbon, which increases the cost of production.

##### Graphene-Based Nanocomposites

There are also hierarchical nanocomposites of vanadium oxide (V_2_O_5_) thin film anchored on graphene, which serve as high-performance cathodes for LIBs [75]. The V_2_O_5_-graphene nanocomposite was synthesized via the slow hydrolysis of vanadyl triisobutoxide on graphene oxide followed by thermal treatment. The authors obtain a specific capacity of 243 mAh g^−1^, 191 mAh g^−1^, and 86 mAh g^−1^ at a current density of 50 mA g^−1^, 500 mA g^−1^, and 15 mA g^−1^, respectively. After 300 cycles at 500 mA g^−1^, the composite cathode exhibited a specific capacity of 122 mAh g^−1^ (64% of its initial capacity). In work [76] Liu et al. showed also V_2_O_5_-graphene nanocomposite as the cathode prepared by adjusting the solvothermal solution. The nanocomposite can deliver specific discharge capacities of 133, 131, and 122 mAh g^−1^ at 16 C, 32 C, and 64 C, respectively. Moreover, the electrodes exhibit a coulombic efficiency of 85% at 1C rate after 500 cycles.

##### LiF-Fe Nanocomposites

In work [77], Li et al. exposed LiF-Fe nanocomposite as a high capacity conversion cathode for LIBs. The authors synthesized material via a simple route of mechanical ball-milling of lithium fluoride and iron using TiN nanoparticles (grinding powders). The cathode delivered a high reversible capacity (568 mAh g^−1^ at 20 mA g^−1^) and it showed strong power capability (300 mAh g^−1^ at 500 mA g^−1^).

##### VOxNTs-Polyaniline Nanocomposites

Vanadium oxide nanotubes (VO_x_NTs)-Polyaniline nanocomposite was used as a cathode material for LIBs and then synthesized by hydrothermal treatment and a wet-chemistry method. The cathode deliviered a higher specific capacity (321 mAh g^−1^ after the first cycle at 50 mA g^−1^) and better cyclig than pristine vanadium oxide nanotubes. The authors summarized that good conductivity and buffer properties of polyaniline as well as lithium storage property and the effective removal of organic template played a critical role [78]. Equation (9) proposed the discharge process for VO_x_NTs:

(9) $$VO_{x}NTs+yLi^{+}+ye^{-}↔Li_{y}VO_{x}NTs$$

##### Carbon-Polymer Composites

In cathode materials, PANi, polyacetylene and PPy can both be applied. The use of conductive polymers is crucial due to their high cyclic reversibility, lower self-discharge rate, and ease of film making [79]. Binary CNT/PANi composites used as positive electrodes showed a Columbian efficiency up to 99%, while the polymer itself showed a maximum current efficiency up to 95%. The specific capacity of the discharge is 122.8 mAh g^−1^ with a current density of 20 mA g^−1^. PANi without composite reaches a maximum of 98.9 mAh g^−1^. The described composite was synthesized by in situ chemical oxidative polymerization. The use of CNT increases the cyclic reversibility, while the composite form shows a much lower load transfer resistance [80]. 

Additionally, the ternary composites play an important role as cathodes. Carbon-LiFePO_4_/PANi composite cathode. The active carbon-based polyaniline composite was introduced to the LiFePO_4_ cathode by chemical oxidation in order to improve the low theoretical capacity of 170 mAh g^−1^. In comparison with the carbon/LiFePO_4_ electrode, the carbon-LiFePO_4_/PANi composite shows 26% capacity enhancement at 10 C [81]. In work [82], LiNi_0.5_Mn_1.5_O_4_-Carbon-poly(3-Hexylthiophene) (LNMO-Carbon-P3HT) composite was used as the cathode. Regioregular P3HT shows amazing self-organizing and electronic properties, which influences the creation of homogeneous nanostructures. The synthesis was carried out by introducing specific functional groups (Grignard metathesis). The specific discharge capacity for LNMO-CNT-P3HT is up to 145 mAh g^−1^, which is much more than without the use of a polymeric compound. After 40 cycles, there is a high Columbian efficiency of up to 80% for composite with polymer, while, without polymer, only 78%, which means that it has improved significantly. Interesting properties have carbon-polymer-sulfur composites. It is caused by the extremely high specific capacity of sulfur (S) 1672 mAh g^−1^ and its environmentally friendly, low-cost properties. Despite great specific capacities, the ionic and electric conductivity is still low, and the presence of dissolution of polysulfide intermediates into electrolyte and changes in sulfur volume limits its applicability. For example, in work [83], Huang et al. exposed a dual core-shell PPy/S/MWCNT nanocomposite as an electrode for lithium-sulfur battery. Carbon material is applied to absorb polysulfide intermediates to some extent and as a conductive network for S. PPy is used to prevent the previously mentioned intermediates from escaping from the sulfur cathode and stabilize the material. Moreover, it is actively involved in the intercalation of lithium ions. The composite exhibits a high specific capacity reaching 1210, 1060, 860, 735, and 665 mAh^−1^ with a current density of 200, 500, 1000, 1500, and 200 mA g^−1^, respectively.

##### Nanocomposites with Self-Assembled Conductive Carbon Layers (CCL)

**Figure 7:**
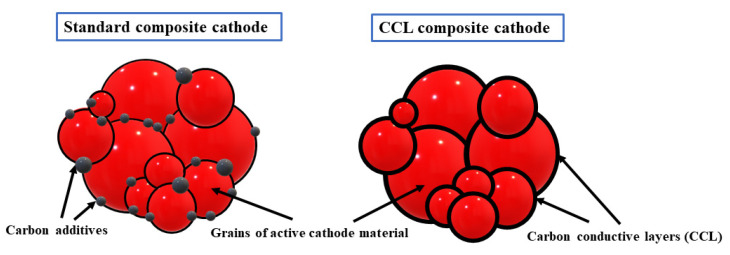
Comparison of standard composite cathode and conductive carbon layers (CCL) nanocomposite cathode in LIBs, based on [84].

According to the manuscript [84], CCL layers form a 3D conductive network, which could absorb volume changes of nanomaterial by the binary structure, increase the chemical stability of nanomaterial, ensure an easy diffusion path, and increase the macroscopic electrical conductivity of nanocomposite (Figure 7). The authors obtained, for Li/Li^+^/(CCL/LiFePO_4_) system, a specific discharge capacity of 163 mAh g^−1^ at C/5 after the first cycle and 172 mAh g^−1^ after the ten cycle, which means that, after first cycle, there is a huge fade of capacity between charge and discharge and after the tenth cycle the capacities of those processes remain almost the same. 

### 2.4. Sodium-Ion Cells

If anyone is interested in energy storage technologies, they certainly know that the current batteries have more than one problem. Not only are they too low in energy density for the needs of today’s world, but they are also very expensive. This is due to the poor availability of metals, such as lithium and cobalt. Sodium-ion batteries not only replace lithium with one of the most popular elements of the Earth, but also give real hope for completely getting rid of cobalt from the electrodes. Sodium-ion batteries are not entirely new. Work on this type of cells has been going on for a long time. The problem is that their results have not been very encouraging so far. First of all, the existing designs did not achieve capacities that were similar to LIB, and what is worse, their service life was poor. Batteries of this type work in a similar way to their current competition, two electrodes are immersed in an electrolyte (there containing lithium salts, here sodium), between which sodium ions jump. Unfortunately, inactive sodium crystals tend to accumulate on the cathode, as a result of which the battery clearly loses its efficiency after several dozen cycles. 

A cathode that was covered with many layers of metal oxides was used and an electrolyte with a much higher concentration of sodium ions was used. This made the ion exchange more fluid, which, in turn, reduced the crystal precipitation process. As a result, the newly tested design retained 80% of its capacity after 1000 charge and discharge cycles. Scientists are now focusing on carefully studying the interactions between the electrolyte and the cathode to fully understand what processes take place there. Despite the fact that, at first glance, the results of this work are not particularly spectacular, the development of stable and cheap sodium-ion batteries can be a real “game changer”, turning the battery market upside down. Of course, this does not mean that cells with a higher density are not needed, but, perhaps in the near future, it is the ease of production and low price that can change the more and more “electric” world for the better.

Lithium-ion batteries (LIBs), with their high energy density, have been widely applied in electronic devices and electric vehicles, but the use of lithium is plagued by high cost and limited resource [85]. Sodium-ion batteries (NIBs), which have wide reserves and low precursor cost, are highly regarded alternative to LIBs, which have been proposed due to its great sustainability without sacrifice in electrochemical performance. Sodium is available over 1000 times more abundant than lithium and it is low-cost. Just as in LIBs, the lithium ion was the carrier, and here the sodium ion is. For the material to serve as an anode, it must meet the following conditions: an atom has to have a low atomic weight, low density, and be able to accommodate vast quantity of sodium ions per formula unit having good cyclability in order to yield stable and high volumetric and gravimetric capacities. Moreover, it has to have a potential as close to that of pure sodium metal, not to react or show any dissolution tendency in the solvent of the electrolyte. Additionally, it must be environmentally friendly and low sourcing. Recently, well known are anode materials, including carbon-based materials, conversion, conversion/alloying, and organic materials studies. Positive electrodes determine the energy density, voltage, and rate capability of a full cell, which are mainly limited by the theoretical capacity and thermodynamics. Those materials have to be highly stable, rapidly react with sodium ions, be a good electronic conductor, and should store a large number of Na ions [86]. In Table 4, some applications of nanocomposites as electrode materials were placed.

### 2.5. Supercapacitors

Supercapacitors are new energy storage devices that exhibit unique features, such as high capacitance, high power density, and a long cycle [102]. Supercapacitors have a very high power density of 1500 W kg^−1^ and above [103]. The storage mechanism is connected with charging and discharging of electric double layer (EDL) and redox reactions. 

#### 2.5.1. NiO-TiO_2_ Nanocomposites

Anandhi et al. characterized a preparation and capacitive behavior of NiO-TiO_2_ nanocomposite, which was synthesized by the sol-gel method and exhibited a flake-like structure. The electrode showed a high specific capacitance of 405 F g^−1^ at scan rate of 5 mV s^−1^ and capacitance retention after 5000 cycles up to 92.32% [104]. In this nanostructure, NiO increased electronic conductivity incorporated into TiO_2_ (as compared to pure titania) and specific capacitance, cycle stability, energy and power density [104]. In work [105] highly-ordered and well-separated titania nanotube array (NiO-TiO_2_) was prepared by a potentiostatic anodization process. The electrodes exhibit high Coulombic efficiency of 92.3% after 1000 cycles and highly accessible 3D redox reaction sites, thus resulting in a specific capacitance of 46.3 mF cm^−2^ (at 0.5 mA cm^−2^). 

#### 2.5.2. Bi_2_O_3_-MnO_2_ Nanocomposites

In work [106], Singh et al. proposed a Bi_2_O_3_-MnO_2_ nanocomposite as a low-cost, eco-friendly, low-temperature solid-state chemical process, followed by air annealing. As prepared electrode exhibited excellent performance properties: high specific capacitance up to 161 F g^−1^ (at 1 A g^−1^) and superior rate capability up to 10 A g^−1^. The biggest advantage of this system is high Coulombic efficiency after 10000 cycles up to 95%, that indicates promising cycling stability. Those values could be achieved, thanks to perfect synergy of oxides and due to polycrystalline and mesoporous structure of nanocomposite. Additionally, a room-temperature ionic layer adsorption and reaction (SILAR) electroless chemical method have been proposed for synthesizing Bi_2_O_3_-MnO_2_ electrode materials over graphite rod in work [107]. The prepared electrode endows a high specific capacitance (350 F g^−1^ at 10 A g^−1^) (better than that of an components separately). 

#### 2.5.3. Fe_3_O_4_@FeS_2_ Nanocomposites

Fe_3_O_4_@FeS_2_ as iron-based nanocomposite was characterized in literature data [108] and prepared using annealing strategy with monohydrate ferrous sulfate as the precursor. The synthesis process remains as a great challenge, because the proportion of FeS_2_ highly influences the specific capacitance of the electrochemical system, but iron-based materials are ideal faradaic electrodes for supercapacitors devices. The electrode delivered an ultrahigh specific capacitance of 597.1 F g^−1^ (at 3 A g^−1^), thanks to the formation of the junction at the Fe_3_O_4_ and FeS_2_ interface, which increases the charge transfer on the electrode surface and interactions of those components improve the electron transfer.

#### 2.5.4. RuO_2_-Based Nanocomposites

Ruthenium dioxide (RuO_2_) has a high theoretical specific capacitance value (1400–2000 F g^−1^) thus resulting in being extensively recognized as favorable materials for supercapacitor devices. The main disadvantages limiting applications are still: high production cost and agglomeration effects. Consequently, RuO_2_ based nanocomposites have been widely studied to optimize the material cost, increase the charging/discharging efficiency for large number of cycles (>50,000 cycles) through the shortening of ion-exchange passages, increase the durability of the device by upgrading its flexibility, miniaturing of the device to increase its portability and easy handling, and widening the working voltage range of the device [109]. Here, we could distinguish:

❖ RuO_2_-based mixed metal oxide nanocomposites are used to reduce the loading of expensive RuO_2_ resulting in smaller capacitance (NiO/RuO_2_ nanocomposite—specific capacitance of 210 F g^−1^ at 5 mA cm^−2^ [110]; TiO_2_/RuO_2_ nanocomposites—good electrochemical results ~990 F g^−1^ at a scan rate of 100 mV s^−1^ [111]; RuO_2_-Mn_3_O_4_ composite nanofiber-mats exhibited gravimetric capacitance of 293 F g^−1^ at 10 mV s^−1^; RuO_2_/TiO_2_ nano-tubular composite achieved a capacitance as high as 1263 F g^−1^ [112]);
❖ RuO_2_-based conducting polymer nanocomposites used because of tunable electronic properties (RuO_2_/polyaniline exhibited specific capacitance of 708 F g^−1^ at 5 mV s^−1^ [113]; porous PANI–763 RuO_2_ composite with a capacitance 664 F g^−1^ at the scan rate of 5 mVs^−1^ [114]; RuO_2_ based PEDOT-PSS (poly(3,4-ethylenedioxythiophene)-poly(styrenesulfonic acid)) that achieved a maximum gravimetric capacitance of 653 F g^−1^ [115]);
❖ RuO_2_-based activated porous carbon nanocomposites used to achieve an improved conductivity and charge-storage efficiencies (hydrous-RuO_2_ with activated carbon nanocomposite exhibited a specific capacitance of 319.3 F g^−1^ at current density of 1 A g^−1^ [116]; carbon nano-onion-based RuO_2_ composites with the capacitance of 570 F g^−1^ [117]);
❖ RuO_2_-based CNT nanocomposites to improve a chemical stability and mechanical strength and decrease the weight (RuO_2_ nanoparticles/MWCNT with capacitance of 450 F g^−1^ at 10 mV s^−1^ synthesized via the microwavepolyol process and via electrodeposition-sinthesized nanocomposite achieved even 1652 F g^−1^ at 10 mV s^−1^ [118,119]);
❖ RuO_2_-based functionalized graphene binary composites (RuO_2_/reduced graphene oxide nanoribbon composite achieved a gravimetric capacitance of 677 F g^−1^ at current density of 1 A g^−1^ [120]; RuO_2_/graphene monolith attained a really huge volumetric capacitance of 1485 F cm^−3^ recorded at 0.1 A g^−1^ [121]); and,
❖ RuO_2_-based ternary composites (Graphene/RuO_2_/Co_3_O_4_ nanocomposites with a specific capacitance of 715 F g^−1^ at current of 1 A g^−1^ [122]).

#### 2.5.5. Graphene-Gold Nanoparticle-Based Nanocomposites

Graphene-gold nanocomposites were efficiently synthesized in work [123] by Ankamwar et al. using high-energy gamma radiation and the second method: chemically from graphite oxide obtained graphene oxide (used a precursor). The structures are eco-friendly and carry low-cost. Using chemical methods and gamma radiation-synthesized, respectively, nanocomposites as electrodes exhibited a stable specific capacitance (100 and 500 F g^−1^ for scan rates of 5 to 500 mV s^−1^). Additionally, electrodes showed high cycle life with Coulombic efficiency up to 90% after 600 cycles.

#### 2.5.6. Graphene Sheets-Cotton Cloth Nanocomposites

In manuscript [124], the authors prepared a flexible and easy processing electrode using everyday cotton cloth and stable graphene oxide suspension as the ink applying carbonization process. This method is called “brush-coating and drying”. Because prepared 3D nanocomposite exhibited good electrical conductivity, strong adhesion between GNSs and cotton fibers. The supercapacitor system was created using graphene sheets-cotton cloth as electrode and pure cotton cloth as separator. The authors achieved a high specific capacitance up to 81.7 F g^−1^ (at 5 mV s^−1^). This system could be widely used in applications such as: portable consumer electronics, hybrid electric vehicles, and computer backup systems.

#### 2.5.7. Graphene-NiFe_2_O_4_ Nanocomposites

Reduced graphene oxide-NiFe_2_O_4_ (RGO-NiFe_2_O_4_) nanocomposites were prepared using hydrothermal process at room temperature. Synthesized nanomaterials RGO-NiFe (at pH = 10) showed the best capacitive properties of circa 345 mAh g^−1^ (at current density of 1 A g^−1^). It was shown that pH adjusting has a significant impact of the electrochemical properties of this nanocomposite as electrode active material [125].

#### 2.5.8. Graphene-Mn-MoO_4_ Nanocomposites

In work [126], graphene-Mn-MoO_4_ nanocomposite was synthesized using in-situ reduction method. The electrode composed reached the specific capacitance of 302 F g^−1^ at 1 A g^−1^ in large potential window of 1.6 V and a maximum capacity retention of 93.8% at 0.7 A g^−1^. The presence of graphene increased the electrical conductivity. Additionally, the high impact has a contact area between components in graphene-Mn-MoO_4_. 

#### 2.5.9. Titanium Dioxide/Graphene Oxide

In work [127], titania powder was synthesized via the sol-gel method, and its surface was functionalized with *N*-2-(aminoethyl)-3-aminopropyltrimethoxysilane (AAPTS) to increase its adhesion to graphene oxide. In the composite, the TiO_2_ nanoparticles are anchored on the graphene oxide via strong Ti–O–C chemical bonds. Such a strong bond gives the composite resilient strength to facilitate the ordered assembly of TiO_2_ nanoparticles and the formation of a mesoporous structure with a high tap density, enabling the rapid transport of Li ions and electrons within the composite structure, and maintaining a stable mesoporous structure during the discharge/charge process of the resultant LIBs. Based on these advantages of strong bonding and mesoporous structure, the prepared composite demonstrated a superior high rate and cycle performance. The high discharge capacity (370 mAh g^−1^) at a current density of 50 mA g^−1^ is impressive and it is among the highest in comparison with other TiO_2_/graphene oxides.

#### 2.5.10. SnO_2_-Carbon Nanocomposites 

The SnO_2_-carbon nanocomposite was prepared by a liquid phase plasma method and it is used as an electrode material for supercapacitor. The electrode exhibited a specific capacitance of 29 F g^−1^ after the first cycle and 28 F g^−1^ after the second one using LPP duration for 60 min. (scan rate of 10 mV s^−1^) [128]. The authors in work [129] synthesized ultrafine SnO_2_ nanorods-rGO nanocomposite while using a two-step hydrothermal method (using KMnO_4_) and those electrode materials in supercapacitors achieved the specific capacitance of 262.2 F g^−1^ (at 100 mA g^−1^). Coulombic efficiency is equal to 96.1% after 6000 cycles, which indicates high electrochemical performance of the system. In work [130] the porous SnO_2_-Cu_x_O nanocomposite thin film on carbon nanotubes as electrodes for supercapacitor’s electrode was synthesized through the electroless deposition technique. The CNT/SnO_2_-Cu_x_O nanocomposite possesses pseudocapacitive behavior, which reaches a specific capacitance of 662 F g^−1^ (at 1 A g^−1^) and the capacity retention equals 94% after 5000 cycles. 

#### 2.5.11. Polymer Nanocomposites

Electric energy storage is an important problem that must be solved in the near future. There is a need for solutions enabling long-term storage of energy obtained from unconventional, renewable sources (sun, wind, water). We also need warehouses that are capable of quickly, short-term taking over excess energy and quickly releasing it. The currently available warehouses are able to provide high power and energy density at the same time, therefore systems are created in which the role of a buffer that is capable of storing and quickly returning excess energy is played by a supercapacitor [131,132,133,134,135]. There are two basic mechanisms of accumulation energy in the supercapacitor, depending on its structure: processes that are related to the formation of a double layer on the electrode/electrolyte interface for carbon electrodes and the so-called pseudo-capacitive processes in which the accumulation of charge is associated with the transport of electric charges in metal oxides [135] and conductive polymers [135,136]. It turns out that, due to pseudo-capacitive processes, oxide materials and conductive polymers, having a relatively small physical surface (from several dozen to one hundred m^2^ g^−1^), also show high capacitance and energy values. A good electrode material should be characterized by high conductivity, which is associated with the super-capacitor charging and discharging speed, as well as high physical strength, which allows for achieving the high stability of the supercapacitor’s properties with repeated charging and discharging. Hence, the interest in carbon nanotubes, which, in combination with the polymer, improve both the electrical and physical properties of the composite. The literature reports [137,138,139] clearly show that the addition of carbon nanotubes to the conductive polymer significantly increases the conductivity of the composite and its stability, reducing one of the serious disadvantages of the conductive polymer. Carbon nanotubes, since their discovery by S. Iijima and colleagues [140], play a significant role in today’s technology for the production of electronic components, including supercapacitors, thanks to their extraordinary mechanical, electrical, and chemical properties. They are used, for example, in field emitters [141], nanometric electronic devices [142], as components of composites and in many other fields. Carbon nanotubes are used as a matrix for many composites: with poly-pyrrole (PPy), poly (3,4-ethylene-1,4-dioxythiophene) (PEDOT) [143], poly (3-octylthiophene), polyphenylvinylinylene (PPV) [144,145], polyacrylonitrile (PAN), and also polyaniline (PANI) [146,147,148].

The polyaniline core-shell nanocomposite with multi-wall carbon nanotubes, produced by the in situ oxidative polymerization method, and its use as an electrode material for a supercapacitor, have become popular. In a semiconductor composite, the nanostructure is closed in the conductor’s mantle. Such a spatial combination of the filler and the polymer matrix gives much wider possibilities of predicting their interaction and controlling the properties of the final composite [149]. The degree of dispersion of the nanotubes also has a smaller impact on the quality of the product, when compared to spherical and plate fillers. Because the dispersion mainly depends on the orientation of the filler particles, only secondarily on the degree of their separation. In the case of the graphene system in the construction of nano-tubes, most of the properties are oriented along the structure that can be used, as intended, in the composite. The multi-walled nanotubes used in the experiment were selected because of their higher chemical and thermal resistance than in the case of single-walled nanotubes [149,150]. Polyaniline is a very stable conductive polymer with good damping and antistatic properties, being resistant to most organic solvents. However, the disadvantage is their low mechanical strength and the fact that it is difficult to process—it can lose its conductive properties when applying high shear forces or high temperature. The conductivity of polyaniline depends on its degree of oxidation. This can be achieved by an appropriate method and synthesis conditions (oxidative polymerization, electrochemical polymerization, and PANI-HCl conductive salt synthesis) or by modifying the finished polymer (doping with sulfonic acids) [151]. Conductivity in polyaniline occurs along the main chain, statistically arranged perpendicular to the nanotubes (at the ‘core-shell’ structure), which may give interesting effects resulting from the spatial orientation of the nanotubes. Vitreous polyaniline can be easily crushed and combined as dry blends with other polymers in order to facilitate the processing or refinement of the material [152,153]. Covering the nanotube completely with polymer is difficult. Polyaniline is a good material for such purposes, because of its environmental stability and ability to control conductivity by doping. In the process of creating a nanocomposite, it is advantageous to use a compatibilizer that increases the efficiency of coating the surface of the nanotube with polymer. Such an agent can be nonionic surfactant or co-polymerizable monomer. Non-ionic surfactants additionally increase the degree of nanotube dispersion, supporting the breakdown of their aggregates [149,151,153,154].

Among all of the carbonized zinc-based structures, which are popular nowadays, MOF-5 (Metal–organic framework) shows the highest porosity and specific surface area, which enables its potential application as electrode material in supercapacitors [155]. The control of the carbonization parameters is important for the properties of the product obtained. For example, the specific surface area of carbonized MOF-5 depends on the thermal conditions of carbonization and it ranges from 1521 to 2542 m^2^ g^−1^ [156], which affects the electrochemical properties of the material. Therefore, the optimization of such parameters turns out to be of key importance for MOF-5 polyvinylidene fluoride (PVDF) structures. Electrodes with a different ratio of active material (MOF-5) to binding material (PVDF) were tested by analytical methods, such as scanning electron microscopy (SEM) and X-ray diffraction (XRD). The advantage of the obtained electrodes is the possibility of synthesizing MOF structures from recovered substrates (DMF after the distillation and terephthalic acid recovered from PET (Polyethylene terephthalate) waste). Another positive aspect is the ability to recover and reuse carbonized MOF-5 from the spent electrodes.

### 2.6. Nanocomposites for Fuel Cells

**Figure 8:**
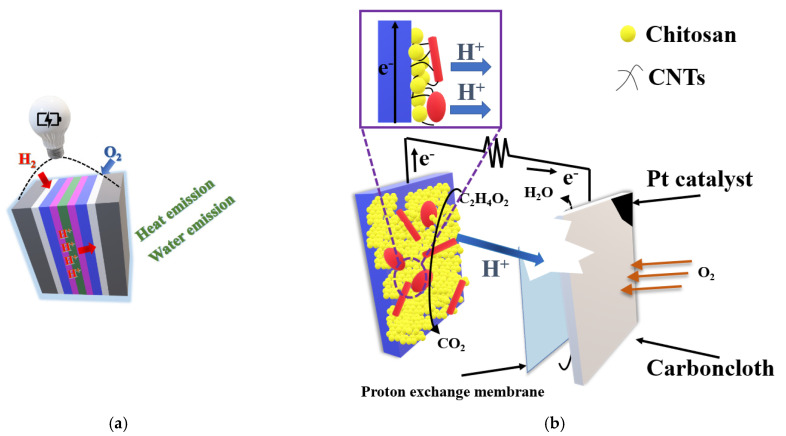
Fuel cell action and structure. The most popular membrane is Proton Exchange Membrane Fuel Cell (PEMFC), which also plays a role of polymer solid electrolyte (**a**); working principles of the microbial fuel cell (MFC) bioanode [157] (**b**).

Fuel cells are one of the many technologies of alternative sources of electricity that has been rapidly developing in recent years. The fuel cell is characterized by one of the highest rates of power yield per unit fuel volume. All without the emission of toxic exhaust components and with very high efficiency of fuel energy use. Taking that modern fuel cells are just entering the development phase into account, the only thing that can really be considered today is not whether, but when they will replace traditional methods of energy production. You can build a power plant that uses such cells literally anywhere and it will work just as well on hydrogen, biofuels, natural gas, alcohol, coal, and many other fuels. Fuel cell systems are widely recognized as the most promising alternative propulsion concept of the future, primarily because of its eco-friendly nature. Fuel cell systems are also considered as future energy sources that are competitive with oil and coal. Fuel cells are electrochemical devices, which transfer chemical energy of the fuel to the electric energy. The cell’s principle of operation is to continuously supply fuel to the anode and air to the cathode (Figure 8a). An electric charge is created on the electrodes as a result of electrochemical reactions. 

In fuel cells, polymer nanocomposites are mainly used as membranes or electrolytes. Polymer nanocomposite has already been proved to be an effective technique for the empowerment of higher temperature and lower humidity fuel cell applications. To satisfy the requirements of the electrolyte membrane for fuel cell, improvements by the application of PNCs were made: water adsorption and retention, ionic conductivity, fuel cell over, thermo and mechanical properties, fuel cell performance, durability, and easier fabrication techniques. 

Some of those membranes are: perfluorinated nanocomposite membranes (with hygroscopic oxides, such as titania or silica; with protonic conductors, such as Zr^4+^ ions; with carbon nanotubes); hydrocarbon membranes, acid-base membranes, and miscellaneous nanocomposite membranes [158]. 

In microbial fuel cells (MFC), carbon-based polymer nanocomposites are often used as electrodes. The utilization process of these materials is a challenge, because there exist some specific characteristics that are hard to fulfill and are very distinct. However, there exist some materials, which could facilitate microbial adhesion and electron transfer, i.e., graphene, carbon nanotubes (CNTs), and conducting polymers, such as poly-N-isopropylacrylamide (PNIPAm) and polyaniline (PANI). It is known that the addition of polymer/carbon nanomaterials can increase the surface area and, therefore, improve the position where bacteria can attach to the electrode surface [159]. Carbon materials, like brushes, graphite, paper, and carbon rods, have all served as anodes in early MFC studies [160]. Generally, they need improvement in biofilm formation, which could be done by increasing the roughness and surface area. It applies to both anode and cathode materials. In general, as anodes we can distinguish: carbon nanotube-modified, polymer-modified, polymer nanocomposite-modified, metal oxide nanocomposite- modified anodes; carbon-based metal-free, carbon-metal-based cathodes. Figure 8b shows chitosan as a commonly used material for enhancing the adhesion of microbes on anode surface, which could produce CNT-chitosan nanocomposite by electrodepositing of chitosan and CNTs together onto a carbon-paper electrode. The power and current densities of MFCs with CNTchitosan anodes rose by 65% and 23%, respectively [161]. However, some of such electrode systems were applied in fuel cells and they are collected in Table 5.

The problem with fuel cells, on the other hand, is aging. The contaminants in the fuel gradually clog the porous electrodes, which inevitably restricts the flow of hydrogen and oxygen ions, reducing current efficiency. Designers try to create sets with a lifetime of no less than 40,000 h (which will mean the necessity to replace the entire block every 5–7 years). An important factor limiting the development of this modern technology is the inexorable economy. Building a conventional power plant is significantly cheaper than a fuel cell plant. Additionally, without orders for fuel cells, manufacturers cannot launch their mass production and, therefore, cheaper and automated production. The interested companies estimate that starting the production of cells with a total capacity of 200 MW per year would reduce their retail price by half. It is worth realizing how huge resources are required in order to remove sulfur and nitrogen oxides from exhaust gases in conventional power plants. Fuel cells do not produce such pollutants at all, and the emission of carbon monoxide is lower than its content in the atmospheric air. Therefore, perhaps it would be more profitable for investors to invest in refining the technology and launching mass production of fuel cells, rather than allocating huge resources to refining old and developing new methods of flue gas cleaning in conventional power plants. Fuel cells have another feature that is difficult to consider in cold cost-benefit calculations: it is a practical solution that stimulates our imagination. Many specialists see them as another important, ecologically clean source of energy in the 21st century [174,175,176].

### 2.7. Solar Cells

**Figure 9:**
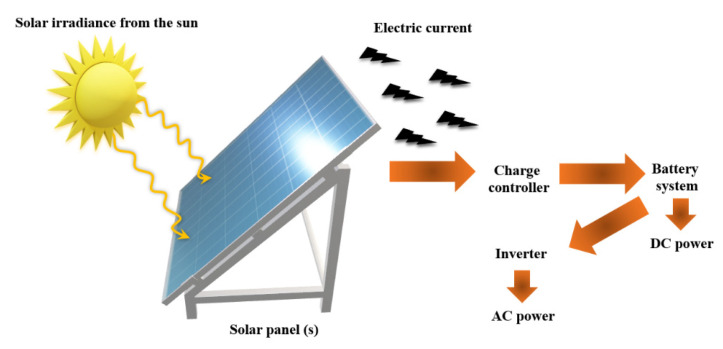
Classic solar cell structure and current production.

Solar cell, which is also known as PV (photovoltaic) cell, converts sunlight energy into the renewable nature of electrical energy by the PV effect (Figure 9) [177].

The photovoltaic effect is the direct conversion of incident light into electricity by a pn (or p-i-n) semiconductor junction device [178]. Generally, solar cells are classified as first (conventional), second (include direct band gap semiconductors), and third (solution-processed)-generation solar cells.

Recent developments of electrode materials include the use of materials based on carbon, metal oxides, polymers, and nanocomposites. These types of electrode materials were greatly enhanced the power conversion efficiency (PCE) of the solar cells [179]. Additionally, in this branch, researchers examine new possibilities of use of nanocomposite materials as electrodes. In Perovskite solar cells (PSCs), some hybrids have also been recently used. Hybrid organic/inorganic nanocomposites are also used in photovoltaic cells. For these materials, some aspects should be taken under consideration to enhance the electrochemical performance of photovoltaic cells. These are the charge transportation along both components, the interfacial area and contact between inorganic and organic components determine the charge separation efficiency, and the energy-level alignment at the interfaces. Moreover, there are is the right combination of inorganic and organic semiconductors. Additionally, nanostructures should be used to provide a large interface for the enhancement of the charge separation process and good contact between organic and inorganic components should be considered. Last but not the least, a nano-structured network of a conducting polymer in the hybrid system is important, because the mobility of ions in conducting polymers is more limited when compared to inorganic materials [6]. The first hybrid electrode was BC-PSCs using transparent QIDEs. Some of them are placed in the Table 6.

The advantages of organic solar cells with a heterogeneous collective junction (BHJ) are of interest in next-generation solar cell applications. There are already electrodes for the practical manufacture of polymeric solar cells (PSCs) while using the optical properties of the electrode material (their operation was tested in PSC devices). Metal electrodes with high performance and improved optical properties, such as aluminum (Al), silver (Ag), and gold (Au), are considered in the manufacture of PSC devices. They consist of a blend of poly (3-hexylthiophene) polymer (P_3_HT) and phenyl-C61-butyric acid methyl ester (PCBM). Among the key photovoltaic parameters, the so-called optical properties had a strong influence on the open circuit voltage (Voc), upper electrode. The increase in VOc of the Al and Ag electrode devices was found to be approximately 11.32% and 26.42%, respectively. These values were much higher when compared to the Au electrode, because the parasitic absorption of the incident photons was below 600 nm. The Ag electrodes have excellent weather resistance, being comparable to Au. The inclusion of monodisperse ZnO as an n-type buffer layer was also analyzed to efficiently transport electrons from the active layer to the cathode electrode [191]. In work [192], the authors compare the environmental stability of methylammonium lead iodide perovskite (MAPbI_3_) solar cells, which contain inorganic ZnO nanoparticle-based hole-blocking layers (HBLs), with reference devices that use the commonly used batocuproin (BCP) as the HBL. While both types of devices exhibit similar initial photovoltaic (PV) efficiency, inorganic HBL is effective in blocking the mobile iodide ions from reaching and reacting with the metal electrode. This does not apply to devices with organic HBL, in which X-ray photoemission spectroscopy (PES) detects a significant amount of iodine. Electrochemical analyzes for photovoltaic cells are most often performed using the cyclic voltammetry (CV) method. The CV method is a very useful on and widely used method of assessing the suitability polymers for applications in organic photovoltaics. The determination of polymer HOMO-LUMO levels by means of cyclic voltammetry facilitates photovoltaic cells with appropriately selected energy levels, which makes it possible to receive cells with a high degree of light energy conversion solar energy for electricity. Sample curves in the CV of the most commonly used organic compounds in the active layer of a polymer photovoltaic cell, i.e., poly(3-hexylthiophene) (P3HT) and a methyl ester[6,6]-phenyl-C61-butyric acid (PCBM), including with an energy diagram of a cell regarding architecture ITO/PEDOT:PSS/P_3_HT:PCBM/Al and the efficiency of PCE equal to 2.9% are presented in paper [192]—zinc and indium oxide (Indium-Tin-Oxide); PSS (polists-rhenium); PEDOT (poly(3,4-ethylene-1,4-dioxythiophene) pentane-2,3,6,7- dibenzoanthracene). Over the last 10 years, there has been an increase in teering with the possibility of using the CV method in the process of obtaining by means of electro- chemical of polymers for photovoltaic applications. Electropolymerization is an alternative to the synthesis of conductive polymers. It is related to connecting longer and longer chains during the passage electricity. After crossing the border, permeability, the polymer precipitates out of the solution and, in the film, builds up on uneven surface electrodes. The most common for describing electropolymerization [193] the mechanism proposed by Diaz [194], which includes three stages: electro- day, merging the mers and deprotonation. Primary advantage electropolymerization is the synthesis of a polymer directly at the electrode, allowing for tight control of the pa-polymer layer thickness, such as thickness [195] or morphology (e.g., in the process of creating nanostructures [196,197,198,199] or nanocomposites [200,201,202,203,204]). One of the more important factors affecting polymer photovoltaic parameter values solar cell, i.e., efficiency and ratio Fill (FF) is the thickness of the layers produced organic (active layer and transport layer hole (HTL)), depending on the size of the transferred charge during electropolymerization. The morphology of the surface of layers that are produced by the electroporation may be controlled by the selection of mical composition of the solution, electrode morphology, and voltage changes over time. The use of electropolymerization also enables the production of nanocomposites polymer cites with the participation of oxide nanoparticles, carbon salts, or nanostructures [200,201,202,203,204]. Electropolymerization is used in both polymer photovoltaic cells (POF) and dye cells (DSSC). Polymer photovoltaics occupies a special place in the development of renewable energy technologies (RES). Over the past five years, we have managed to increase the efficiency of the obtained cell (PCE) with 5.15% (year 2010) to 13% (year 2012) [205]. Photovoltaic cells limers are the links of the third and fourth generation. The cells of the third generation should be distinguished as organic, both polymeric and compound-based low molecular weight cells and dye cells. *In turn, the fourth generation cells are mostly organic groups, including polymers, chemically modified oxide graphene, nanotubes, TiO_x_ or ZnO* [206,207]. Work on the development of polymer photovoltaics, which was carried out in Wrocław (Poland), aimed at the construction and characteristics of photovoltaic cellular polymer cells, both on the substrate rigid as well as flexible, containing polymer rye of various chemical structure, fullerene derivatives (PCBM—[6,6]-phenyl-C_61_-butyric acid methyl ester, PC_71_BM—[6,6]-phenyl-C_71_-butyric acid methyl ester), as well as graphene oxide, nanotubes, TiO_2_, Ag, and liquid crystals [208,209,210,211,212,213,214,215,216,217,218,219,220,221]. Details of the mechanism of action and type of organic photovoltaic cells is discussed in the publication [206,222].

### 2.8. Nanocomposite Application in Flexible Energy Storage and Generation Device Application

Energy storage is a critical technology for most defense and commercial applications to use energy in an efficient manner. Much attention is paid to flexible energy storage sources, due to the strong need for miniaturization of devices, including flexible displays and portable electronics. Some energy storage devices, such as electrochemical capacitors (or supercapacitors), metal ion batteries, and, more recently, metal air rechargeable batteries, have been identified as the most practical and feasible technologies. The development of flexible electronics urgently requires lightweight, able to be rolled, and flexible energy storage devices that have high power and energy density. Unfortunately, the major limitation are ecological aspects, volumetric energy density, high internal resistance at the interface between materials, and poor mechanical strength. In order to overcome these problems, electrode materials with high volumetric and surface capacity are used. It also aims to reduce the internal resistance and reduce the quantity of inactive materials in the electrode paste. Moreover, the ionic conductivity of the electrolyte foil should be increased by doping when all-solid-state electrolytes are used. The most important aspect is the drive to use the nano-scale to increase material efficiency.

The main energy generating systems are solar and wind sources (DS-PECs, Bio-PECs, OIHPSCs, and OSCs). Flexible dye-sensitized photo-electrochemical cells (DS-PECs) are used, due to cheap production costs, readily available raw material, high efficiency, and ecology of production. They are mostly used as materials for the production of solid electrolytes to improve work safety. Most significantly, SOICs devoid of additives (i.e., single component) exhibited high charge mobility and conductivity. Novel SOICs can be synthesized with a stable organic radical (e.g., 2-azaadamantan-N-oxyl (AZA), 2,2,6,6-tetramethyl-1-piperidinyloxy (TEMPO)) instead of an unstable iodide radical as a solid-state electrolyte, due to the organic radical being a potential redox mediator with furnished dual channels for easy charge transfer [223].

In lithium-ion cells, which have excellent volumetric and gravimetric energy density, layered materials are often used when creating electrodes, which are involved in the intercalation/deintercalation of lithium ions during charging/discharging. Despite the volume expansion during the action of lithium, they show less energy storage capacity. In order to overcome this problem, carbon materials are used to form a flexible, high conductivity network. Examples of such materials are NiCo_2_O_4_ carbon fiber anodes, ploypyrole composite/porous silicone hollow spheres. Polymer coatings significantly improve the conductivity of the electrode and stabilize the structure. When creating flexible cells that consist of Mn_2_O_3_ (anode) and LiMn_2_O_4_ (cathode) nanowires, the transport path of lithium ions is shortened. A big problem with these cells is also: dendrite formation, electrolyte leakage, sudden temperature increase, low resistance to temperature conditions, and it solved by using ceramic separators (i.e., pure aluminum oxide nanowire-based membrane). Additionally, co-axial nanowires/nanotubes are used because of their multiple functionalities by combining the physical and chemical properties of different materials (i.e., 1D tin oxide core and indium oxide shell, MnO_2_-CNT nanohybrid). The co-axial morphology offered a unique combination of high porosity and low internal resistance. In order to improve the safety aspect, coat polymeric materials and add functionalizations may be very useful in controlling secondary electrolyte interphase formation while using nanostructured materials. The use of ionic liquids turns out to be a good solution and, by adding nano-sized ceramic fillers to solid electrolytes, the efficiency is increased. In magnesium-ion, sodium-ion, and aluminum-ion cells, the main disadvantage is the slow diffusion of metal ions. A good solution is the production of 3D electrodes, but the still high price and complicated production methods are block their development. Such electrodes provide excellent electrolyte wettability, fast electrical conductivity, and a high level of sodium ions in flexible energy sources [224]. Various multifunctional hybrid nano-structured materials are currently being investigated to improve the energy density and power of next-generation storage devices. Templated hybrid nanostructures, such as flexible films of CNT/AuNW hybrid structures, are used in supercapacitors, which increase the stability and power density (low contact resistance) many times thanks to nanotubes [225]. Additionally, work [226] has shown that flexible energy storage devices could be based on nanocomposite paper. Nanoporous cellulose paper embedded with aligned carbon nanotube electrode and electrolyte constitutes the basic unit. The units are used to build various flexible supercapacitor, battery, hybrid, and dual-storage battery-in-supercapacitor devices. They are used to ensure the flexibility during the work of storage device. Moreover, the discharge capacity and performance observed here compare well with other reported flexible energy-storage devices, which makes them appropriate for future energy storage devices.

Finally, safety aspect should be a priority when developing flexible energy equipment. For example, flexible supercapacitors provide low energy, hence the importance of developing flexible batteries. An extremely important aspect when improving the functioning of flexible energy sources is: the integration with other energy sources, creation of biocompatible materials, implementation of new nano-production techniques, and construction of new storage devices [224].

Currently, houses are being designed to use these sources, e.g., houses with solar collectors. The resources of coal and crude oil are decreasing and the costs of transporting these fuels are rising. Environmental pollution and the penetration of carbon dioxide into the atmosphere are also increasing, which causes, for example, severe weather anomalies, hurricanes, and floods. Energy consumption is currently increasing significantly, which leads to a greater focus on alternative energy sources and cost optimization in terms of energy production itself.

### 2.9. Safety of Energy Carriers

Research on individual components and entire systems of lithium-ion cells is becoming increasingly popular in the world of science. This is due to the deep belief that the development of these batteries is able to satisfy the constant needs of consumption of society in any place and at any time. Because of the large number of parameters determining the electrochemical properties of a lithium-ion cell, the introduction of a new component into it (e.g., an electrolyte salt or an electrode material) often changes all of the processes taking place in the battery during charging and discharging. It is this interaction of individual components of the cell that causes difficulties in describing the effects that accompany attempts to modify them; on the other hand, it makes it possible to search for an ideal system in which the synergistic effect of interaction of individual battery components results in a system that is characterized by excellent properties. During charging and discharging, lithium-ion cells engage in a reversible insertion/deinsertion of lithium ions into/from a matrix of electrode materials, called intercalation compounds

Like any element belonging to the group of alkali metals, lithium is chemically unstable in the presence of all known non-aqueous electrolytes, which results in a significant reduction in the safety of using a battery containing it. One of the directions of research was the use of the lithium bonding strategy in the intercalation material, such as on the side of the positive electrode. The system in which both the anode and the cathode contain intercalating materials has been called a “rocking-chair cell”, because the lithium ions are “swinging” between the positive and negative electrode arrays.

A lithium ion cell is made up of three main components, i.e., the addition electrode, negative electrode, and electrolyte. Note that each of these components works at the limit of thermodynamic stability. It often happens that, in a battery, this stability is often achieved by creating a dynamic balance between its individual elements. Therefore, the design of new rechargeable lithium-ion battery systems involves the need to predict the interaction of electrode materials and the electrolyte. Currently, commercial technologies of materials that are used in lithium batteries are confined to two families of negative electrode materials (carbon materials and lithium-titanium oxides—LTO), five families of positive electrode compounds (lithium-cobalt oxides—LCO, lithium-manganese oxides—LMO, oxides lithium-nickel-manganese-cobalt-NMC, lithium-nickel-cobalt- aluminum oxides-NCA, and iron-lithium phospho-olivins-LFP) and three families of electrolytes based on various lithium salts dissolved in organic carbonates. This gives the potential to construct 40 systems with different electrochemical properties, which significantly extends the applicability of lithium-ion cells in various areas that require energy storage [227]. Unfortunately, many of these theoretical combinations of components are impossible to use in practice due to the lack of establishing the previously mentioned thermodynamic equilibrium between them.

Durability is expressed in the number of charge/discharge cycles, and the end of battery life is usually taken as a drop in capacity to 80% of the nominal value. The durability of a lithium battery depends on a number of factors, which includes operating temperature, storage temperature, and the course of the charging process [228].

In order to increase the durability, it is recommended to store unused Li-Ion (Li-Poly) batteries at a relatively low temperature (about 6 °C) and, quite importantly, preferably only charged up to 40% of their capacity. The specific operating temperature limits depend on the manufacturing technology of a given cell variant. According to the rules governing chemical reactions, the current efficiency and effective charge of a lithium cell decrease as the temperature drops. Using the cell below the temperature of –20 °C is possible, but its energy properties will be very limited, and its durability will be violated. In winter conditions, the concept of a heater seems to be justified, as it will improve the working conditions of lithium cells that make up the battery, but, by consuming energy, it will reduce the service life [229]. Temperatures that are above the optimum are not recommended for safety reasons, especially in a situation where the battery consists of hundreds of cells and their cooling is limited and unequal. Already slightly above the limit temperature of a given type of cell, significant deposition of metallic lithium occurs. If the temperature rises significantly above the limit temperature, the passive layer separating the negative electrode and the electrolyte may be broken. This leads to a strongly exothermic reaction of graphite with the electrolyte and it can lead to pressure increase and cell swelling or gas discharge through the safety valve. Therefore, if free oxygen is found in the cell, e.g., from the decomposition of the cathode matrix caused by heat, the damaged cell may ignite and/or explode. In order to equalize and lower the temperature of cells in the battery, it is possible to use a forced liquid or air-cooling system. The influence of temperature must also be taken into account when charging the battery. As its value decreases, the permissible voltage decreases, above which the risk of formation of metallic lithium at the anode is high. Manufacturers of lithium cells do not recommend charging at a temperature below 0 °C or order charging with low currents of C/10, which necessitates a long process time.

**Figure 10:**
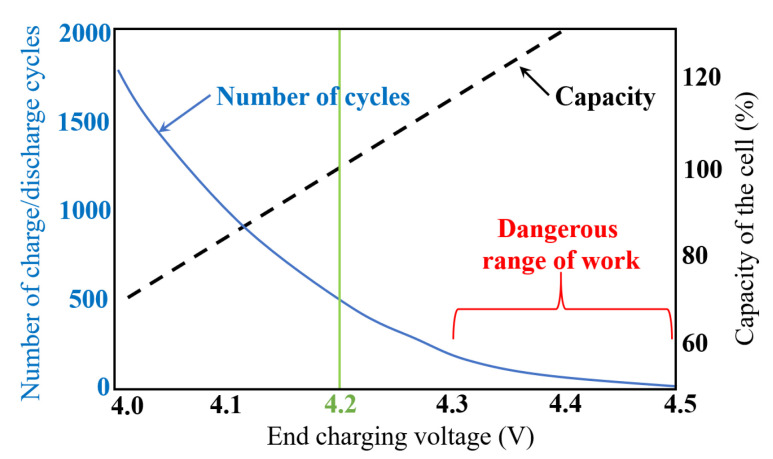
Dependence of service life on final charge.

Charging a lithium cell takes place in two phases: at a constant current (CC) when the cell voltage is even lower than the value that is considered to be acceptable at a given temperature, and at a constant voltage (CV) and decreasing value current. Of course, the charging current also cannot exceed the permissible value for a given temperature that is specified by the cell manufacturer. Charging a cell is an important stage of its operation and it has a large impact on its durability [228] (Figure 10).

**Figure 11:**
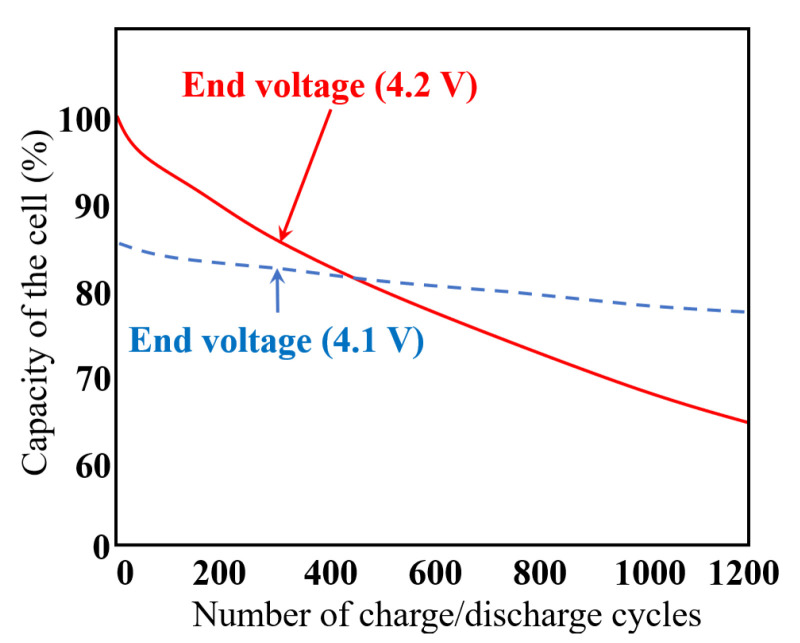
Extending battery life by lowering the end voltage value.

The most common voltage is 4.2 V as the end voltage for charging lithium-ion and lithium-polymer batteries (but lower in relation to iron-phosphate LiFePO4—usually 3.6 V), while the value of 4.2 V is not the optimal value, only kind of a compromise. Thus, the end voltage may be in the range from 4.0 V to at least 4.3 V. The lower the end voltage, the smaller the resulting capacity, of course. However, lowering the final voltage significantly increases the service life (Figure 11).

Hence, if, for the rated voltage of 3.2 V, the durability is estimated at about 2000 work cycles, then at the end voltage of 4.1 V it is twice as long. On the other hand, the capacity does not decrease dramatically, as it is around 84% of the rated capacity. On the other hand, increasing the end voltage to 4.3 V causes a serious, more than two-fold reduction in durability to about 1000 cycles. The capacity increase is only 15%. Thus, when the end voltage is reduced by 0.1 V, the loss of capacity will be small, at most 15%, and the service life extension—large, more, or less twice (Figure 11).

**Figure 12:**
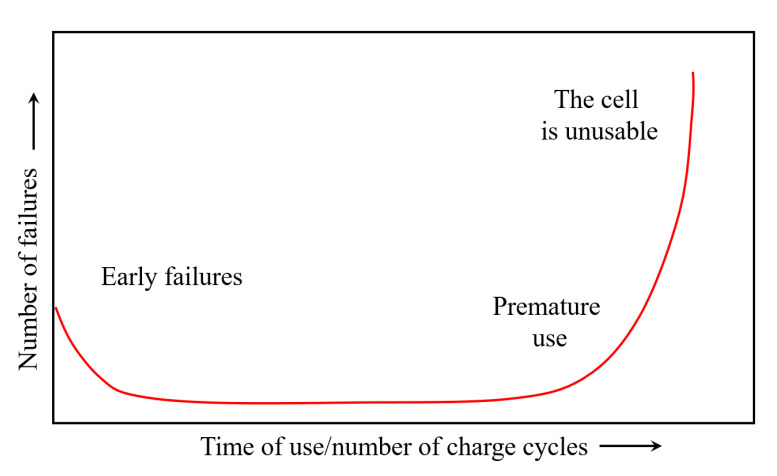
Charge profile.

Lowering the end voltage also slows down the loss of capacity (Figure 12). After 450 cycles, the battery only charged to a voltage of 4.1 V will have a capacity greater than that of a charged voltage of 4.2 V. Unfortunately, not all manufacturers provide this type of detailed information, and some companies’ products may have slightly different properties than those described here. However, it should be noted that the end voltage of 4.2 V is a compromise between capacity, durability and, in part, the duration of charging.

The main cause of early revealing battery failures is, above all, errors in their construction. They are caused by design errors or the omission and installation of damaged or missing components in the device. The latter may, for example, have the wrong dimensions. If they are not made of a suitable material, then it is possible that, in turn, they will be less mechanically strong and less resistant to corrosion. Poorly finished components are also a problem. For example, sharp edges can damage the electrode separators, causing an internal short circuit of the battery.

The impurities that are introduced into the interior of the energy storage during production are also a serious problem, which react with the materials and chemicals used to build the battery (Figure 12). New compounds that are formed by various chemical reactions diffuse in the electrolyte, eventually depositing on the electrodes. In addition, some reactions can be very violent. This causes the internal pressure of the accumulator to increase. Problems, such as weak connections in the structure of the reservoir and leaks in its housing, are also revealed early on. The resulting electrolyte leakage and the ingress of moisture into the interior of the device are reflected in its lower performance. The battery may also wear out prematurely for various reasons. This is mainly due to the gradual change in the properties of the active materials. Other reasons include the progressive destruction of materials used to make separators and device seals.

The battery also wears faster as a result of self-discharge, i.e., the spontaneous discharge of the reservoir that is not connected to the electricity receiver. Batteries are built in such a way that the chemical energy that is stored in them is gradually released in the form of electricity supplied to consumers. Uncontrolled and violent release a causes temperature increase that may lead to fire or explosion. The debris from such an explosion may injure people in the immediate vicinity or damage neighboring devices. In the event of a fire, the receiver that is powered may also be damaged.

During such events, toxic and corrosive liquids leak into the environment, and various types of poisonous gases are released. Effective diagnostics of battery cell packages is required, so that they can function as a reliable and stable source of electricity for as long as possible, being characterized by high energy efficiency and a high level of safety. Usually, it is implemented using specialized electronic systems known as BMS (Battery Management System), which are implemented for specific battery solutions [230,231,232,233].

The BMS system prevents damage to the lithium cells that make up the battery. It performs a number of functions, such as:

➢ measurement of system voltage,
➢ current and temperature,
➢ cell charge level,
➢ cell protection, temperature management,
➢ controlling the loading/unloading procedure,
➢ data acquisition,
➢ communication with internal and external modules, and
➢ monitoring and storage of past data.

The most important task of this system is to equalize the voltage on battery cells, wich is called balancing (balancing) the cells [229]. The discrepancy in the amount of energy stored in the cells in a battery system is very important in terms of battery life. Without a BMS system, the voltage values of individual cells can vary greatly over time. The capacity of the entire package may also decrease rapidly during its operation, which results in the loss of total battery system viability for further operation.

The accumulator battery consists of selected cells. They are selected in terms of electrochemical properties so that they are the same as possible. Such a selection of cells does not require cell balancing, because the differences in voltage and electric current are small [154]. This method is not sufficient for maintaining a series of series-connected cells in equilibrium throughout the service life. After a longer period of time, there may be significant differences that are related to their self-discharge and different levels of charge resulting from uneven aging of the cells themselves. This method can therefore only be used for selected cells.

Passive balancing is the dissipation of excess energy into heat using properly selected resistors. In this case, the voltage values of individual cells are monitored by the microcontroller through the A/C converter, the input of which, through the multiplexer, is switched on to individual cells. If the voltage value of one of the cells significantly exceeds the voltage of the others, then the appropriate S key is turned on. This results in the discharge of the cell through the passive balancing circuit element—a resistor, connected in parallel with each cell and lasts until the voltage of the overcharged cell equals the voltage value of other cells. Subsequently, loading of the packet continues.

However, passive cell balancing has drawbacks. One of them is low efficiency, resulting from the fact that the excess energy accumulated in unbalanced cells is lost to heat in the resistor. In addition, the total capacity of a set of batteries is limited by the need to adjust the charge level of the cells to the capacity of the “weakest” of them [234,235]. Therefore, passive balancing can only be performed during the charging process. However, this way it is not possible to prevent the imbalance of cells that occurs during their use and which is usually a consequence of the phenomenon of their self-discharge. Nevertheless, overcharge compensation is only effective for a small number of series-connected cells because the difficulty of leveling increases exponentially as the number of cells in series increases. In general, these methods are cost effective solutions for low voltage lead-acid batteries and nickel compounds.

Active cell balancing is an alternative to the passive method. The basic idea is to use an external system designed to actively transfer energy between the cells. The method of active balancing of cells can be used in most modern cells from the lithium group [234,235]. There are many methods of active cell balancing and they are classified differently.

The service life of lithium-ion batteries is also an important parameter, especially for owners of electric cars, because their energy storage properties deteriorate over time. The speed of this process depends on the materials that are used for their construction, their construction, and the way they are used—faster aging is favored by, among others, battery overheating. For example, the service life of batteries used in telephones or laptops is usually only a few years, after which they need to be replaced. Batteries in electric cars must necessarily be more durable—typically their guaranteed lifetime is from eight to 10 years. Nevertheless, their ability to store energy declines over time. This, in turn, has an impact on the reduction in the price of the car in the event of its resale from year to year. For many, this is an additional deterrent to purchasing a vehicle of this type. Various problems reduce the service life of batteries. Examples include: losses of electroactive ions, excessive growth of passivation coatings formed on the electrodes, delamination of the electrode material from the foil, and cracking of the electrodes under the influence of mechanical stress. Concerns about the safety of lithium-ion batteries are expressed by owners of consumer electronics and electric cars. There have been several cases of fires in recent years, in which they played a decisive role.

The alternative methods of obtaining energy include water energy (water wheels), air, wind (wind turbines, windmills), solar energy (solar collectors), geothermal energy coming from the Earth, biomass, and biofuel. Hence, a far-reaching search of researchers is necessary to build cells and create various materials at the same time.

With such an extensive topic, it is worth mentioning that the range of electric cars is gradually increasing, but it is still a significant problem that is related to electromobility. The large capacity of the battery does not solve it, because the issues that are related to the quick replenishment of the energy stored in the batteries remain open. In this context, the importance of other elements in electric and hybrid cars for range and energy saving, including careful use and tires, is not diminishing. Scientists are working on batteries for electric vehicles that will be able to absorb a charge of energy up to a hundred times faster than the currently produced lithium-ion batteries. The problem, however, is the availability of sufficient power sources. The energy used by electric vehicles can be saved in different ways and it does not necessarily involve any sacrifice on the part of the user. Currently, the biggest problem with charging electric car batteries from public energy sources is the time that it takes to fill the batteries. The limitations are on the side of both chargers and cars. Modern lithium-ion batteries are moderately stable batteries—they are sensitive to the increase in temperature associated with the intake and return of energy, and, therefore, require constant voltage and temperature monitoring, as well as cooling. Additionally—most importantly—their ability to accept a charge per unit of time is limited. At the moment, there are few cars that can fully use the power of a charger with a capacity of 100 kW or more. There is probably no hope that batteries based on lithium-ion technology will be able to use public chargers with a capacity of 350 kW in the foreseeable future. Additionally, this is what—the ability to absorb a huge dose of energy in a short time—is the key to solving the problems related to electromobility. If it was possible to charge a car with a range of 500 km in a few minutes, then even a limited number of public charging points would not be an obstacle.

### 2.10. Electrode Degradation

Nowadays, modifications of the existing solutions for the alternation of electrode materials in electrochemical devices are very popular. However, there are many points that need to be taken into account and looked at critically. Many electrodes, despite their excellent electrochemical properties, such as high specific capacitance, are unable to cope with the issues of, e.g., temperature resistance, electrochemical resistance, or poor cyclic stability. This leads to a loss of Coulombic efficiency and the subsequent degradation of the electrodes. For the user of the battery, this may significantly reduce the condition of the battery, as well as increase the flammability of the cell. This topic has been taken up by many scientists [236,237,238,239,240,241,242,243]. The currents flowing through the cell depend on the diffusivity of lithium ions [244]. Systems that quickly achieve the equilibrium concentration of lithium at the phase boundary between the electrode and the electrolyte during the change of the potential applied between the cell terminals, and also with uniform electrodes, conduct a current that is mainly limited by the diffusivity of lithium ions, so the intensity will depend on the diffusion coefficient of lithium ions [245]. Inside the crystal they diffuse mainly through the interstitial positions due to their small radius, but this movement is strongly inhibited by adjacent ions. In polycrystalline materials, the movement of lithium ions at the grain boundaries is also noticeable. The diffusion coefficient of this type of movement is often greater than inside the grains, due to the low activation barriers and high concentrations of defect mediating therein. Along with the diffusion in the lithium-ion cell during operation, the electro-conductivity also coexists, being necessary to supply the charge to redox processes [244]. The rate at which the lithium ions diffuse into and out of the material is also important. The reduction of the material grains, which leads to an increase in the ratio of the exposed surface to their volume, increases the speed of this process, shortening the diffusion path [246]. Diffusion through the interface of the electrode with the electrolyte is also dependent on the grain orientation [247]—certain crystallographic planes expose more channels through which lithium ions diffuse, which is especially important in the case of materials that are characterized by one or two-dimensional conduction paths. The conductivity can also be increased by controlling the microstructure and morphology of the crystallites. Particularly for layered materials, certain grain shapes minimize the diffusion path [248]. During operation, the cells lose some of their capacity. This is often due to the loss of charge carriers, e.g., to the passivation layer deposited on the surface, often of both the graphite anode and the cathode. The service life in these systems also depends on the nature of the interface of the electrodes and electrolyte—it is important that the material resists washing away by, or other interactions with, the electrolyte solution. During the discharge or charging of the cell, the incorporation or incorporation of lithium ions may lead to fracture of the material and breakage of its parts, as a result of changes in network parameters or phase transformations, leading to changes in the volume of the material [249]. This safety results from the stability of the anode, cathode, and their surfaces [241]. The average cell achieves voltage ranges that exceed the windows of the thermodynamic stability of the electrolytes and, therefore, they often undergo exothermic decomposition or redox processes when in contact with both anode and cathode charged active material. Moreover, high potential cathode materials can release oxygen at elevated temperatures [241]. All of the above-mentioned properties and many other parameters can be modified by various treatments. However, it is most often associated with the improvement of some and the deterioration of others. For example, particle reduction results in better conductivity, which is associated with shorter diffusion paths and a larger electrode-electrolyte interface, but, on the other hand, the same fact causes a reduction in the stability of the electrode, increasing the loss of cell capacity during operation due to higher activity surface.

## 3. Conclusions

The use of traditional and innovative nanotechnologies for the production of surface layers and coatings to produce a nanocomposite material with properties that are unattainable separately for both the base material and the surface layer material is now the main goal of surface engineering and improvement of construction materials. Nanotechnology enables the production and use of tools and materials, which show unique properties due to their small size.

Contemporary electrochemistry dispositions of a modern tool, which are modified electrodes, including micro- and nanostructured electrodes. It has a large number of classic redox systems, various types of nanostructures, and a large number of modern conductive materials and matrices. Such a wealth of materials that can be used to build electrodes means that, today, we can talk more and more often about designing electrodes with predetermined properties, intended for specific tasks. At the same time, it should be remembered that the development of new electrodes is one of the fastest developing trends in modern electrochemistry.

There seems to be a growing demand among consumers all over the world for ever lighter, smaller, more efficient, and more powerful batteries that provide better parameters of various devices, e.g., allowing them to generate more power or work longer. This state of affairs creates a need in business to develop better solutions and technologies. Lithium-ion batteries are currently of interest to many research groups from both academia and industry. So far, they are most often used as energy sources in portable electrical devices, and it is expected that, with their development, the group of their applications will expand, among others for electric vehicles, autonomous devices, and much more. Still, many elements of the cells require the solution of various problems, and the field, therefore, leaves a wide choice in the directions of research and improvement associated with such devices. However, progress in it often requires interdisciplinary skills in physics, materials science, electrochemistry, chemical engineering, computer modeling, etc. Nevertheless, the above-mentioned materials promise solutions allowing for the construction of high-power lithium-ion cell batteries.

The temperature range was taken into account in the analysis. For example, in a classic lithium-ion battery at temperatures that are above 65 °C, metallic lithium is deposited. However, above 80 °C, the passive layer separating the negative electrode and the electrolyte is broken, which leads to a strongly exothermic reaction of graphite with the electrolyte and, ultimately, to the complete destruction of the battery. At temperatures exceeding 110 °C, the organic components of the electrolyte decompose with the release of flammable hydrocarbons. This results in an increase in pressure and the cell swelling or gas ejection through the safety valve. If there is free oxygen in the cell, e.g., from the decomposition of the cathode matrix caused by heat, ignition may occur. Above 140 °C, the separator melts and the electrodes are short-circuited. Li-ion batteries should not be charged in temperatures below 0 °C. At 0 °C, the charging current should be limited to 0.1 C, which results in a significant extension of the charging time. When developing new compounds that are contained in a cell, one should not forget about the applicative nature of the research being conducted. The synthesized compounds must be low cost, high purity, and short preparation time. The synthesis procedure itself must also be transferable to an industrial scale. Another important aspect is the degradation of the electrode material during operation. A series of chronoamperometric tests are performed in order to determine the useful specific capacity and cyclic resistance of the electrodes. They showed that the reversibility of the process is limited and it results in a significant decrease in the specific capacity of the electrode in subsequent work cycles. The reason is the mechanical degradation of the electrode structure resulting from changes in the volume of the electrode (e.g., silicon). On the surface of the electrodes, places of delamination of the active material can be observed. The electrolyte then contains visible grains of active material detached from the electrode.

Safe and reliable use of lithium cells requires supervision, parameters, and states of their operation, determination of many quantities characterizing the limits of their use, ensuring cooling and appropriate mechanical protection.

The alternative methods of obtaining energy include water energy (water wheels), air, wind (wind turbines, windmills), solar energy (solar collectors), and geothermal energy coming from the Earth, biomass, and biofuel. Hence, a far-reaching search of researchers to build cells and create various materials at the same time.

In summary, hybrid materials and nanocomposites have found wide application as electrode materials in many energy sources. Functional hybrid materials are of particular use. The use of these materials allows for you to overcome the problems that are related to changes in the structure of materials during electrochemical processes, improve the electrochemical efficiency, and use them in more demanding energy applications. Materials that are based on natural sources are of particular interest, but they show limited conductivity and are still being improved by researchers.

## Figures and Tables

**Table 1:** Some hybrid materials applied in electrochemical devices.

Hybrid System	Description	Specific Capacity of Battery/SC	Limitations	Function of the Hybrid	Electrochemical Device	References
Polypyrrole-lignin	Conjugated polymer/lignin hybrid for scalable energy storage. Stable supercabatteries (combining the merits of battery and supercapacitor). In stationary storage low cost of lignin biopolymer		Stability/lifetime and self-discharge	Electrode	Supercabattery	[11]
CNT-TMO CNT-ECP CNT-polyaniline CNT-PPy	Nano-hybrid material with interfacial conjugation (Π-Π interactions) for supercapacitors.Electronically conducting polymers (ECPs) and TMOs are semiconductors. CNT (carbon nanotube) hybrids create thick electrode films and enable high energy capacity devices		Low conductivity	Electrode	Supercapacitor	[12]
CNT-reduced graphene oxide	Highly stable, electrical conductive, with high mechanical strength hybrids. The use of hybrid system in enhancing the performance of LIBs and supercapacitors to support the large-energy storage devices (electric vehicles)		Structural flaws, defects (lower capacity)	Cathode/Anode	Supercapacitor/LIB	[13]
Polyaniline/PMo12 ABPBI/PMo12	Polyoxometalates in PEM fuel cells, electrochemical capacitors, catalysis, sensors, photoelectrochemical conversion for electrodes and electrolytes. They have high good protonic conductivity. Limitations: lack of adequate cyclability.			Cathode	Supercapacitor	[14]
Lithiated λ-MnO_2_	Hybrid material for supercapacitor’s application: manganese oxide (MnO_2_) from spinel as promising material. The system consists of: lithiated λ-MnO_2_ (cathode) and activated carbon (anode)	60 F g^−1^	At higher current densities and voltage scan range reduction of specific capacitance	Cathode	Supercapacitor	[15]
Li_1.4_Fe_6.8_[CH_2_ (PO_3_)_2_]_3_[CH_2_(PO_3_)(PO_3_H)·4H_2_O	Hydrothermally synthesized lithium iron methylene diposphonate (transition metal) as a new organic-inorganic hybrid cathode material for LIBs. Coulombic efficiency of 97.6%	128 mAh g^−1^ after 200 cycles at 20 mA g^−1^	Low initial coulombic efficiency	Cathode	LIB	[16]
LiFePO_4_– Li_3_V_2_(PO_4_)_3_ LiFePO_4_–LiCoO_2_ LiFePO_4_–LiMn_2_O_4_ LiFePO_4_–LiVPO_4_F LiFePO_4_– LiMnPO_4_ Li_3_V_2_(PO_4_)_3_– LiMnPO_4_ Li_3_V_2_(PO_4_)_3_– LiVPO_4_F Li_3_V_2_(PO_4_)_3_– LiVOPO_4_ LiCoO_2_–LiMn_2_O_4_	Hybrid cathode materials for LIBs in electric vehicles, hybrid electric vehicles. F. e. LFP-LVP hybrid has max. initial discharge specific capacity in equal to 166 mAh g^−1^ at 0.1 C	LFP-LVPF 160 mAh g^−1^ at 0.2 C; LMP-LVP 154 mAh g^−1^ at C/50	Synthesis has many internal and external influential factors, mechanism of mixing process, how to ensure uniformity	Cathode	LIB	[17]
Vanadia–titania	Vanadia–titania multilayer nanodecoration of carbon onions via atomic layer deposition for high performance anode for fuel cell	382 mAh g^−1^ of the composite electrode (554 mAh g^−1^ per metal oxide) with an impressive capacity retention of 82 mAh g^−1^ (120 mAh g^−1^ per metal oxide) at a high discharge rate of 20 A g^−1^	Vanadium dissolution at low voltages	Anode	Fuel cell	[18]
Graphene-vanadium oxide	Hybrid electrodes for supercapacitors. RG (0.5)/VO_x_·nH_2_O electrode with RG content of 10 wt% (60% capacity retention)	384 F g^−1^ at a scan rate of 5 mV s^−1^ after 1000 cycles	Graphene amount	Electrode	Supercapacitor	[19]
TiO_2_-CNTs	3D conductive network hybrid nanostructures as anode materials in LIBs. Mesoporous TiO_2_/CNTs stable capacity retention, high Li storage capacity, superior rate performance	203 mAh g^−1^ at 100 mA g^−1^	Relatively small specific capacity	Anode	LIB	[20]
Manganese dioxide-lignin	Manganese dioxide and lignin activated by ionic liquids-based anode material for LIBs	610 mAh g^−1^ at 50 mA g^−1^ 570 mAh g^−1^ at 1000 mA g^−1^	Non-farradaic reactions MnO_2_/KL+A|Li, MnO_2_/KL+B|Li systems (absence of oxidation and reduction peaks – KL-kraft lignin)	Anode	LIB	[21]
TMOs-carbon Ti-based TMO Nb-based TMO Fe-based TMO Co-based TMO Ni-based TMO Cu-based TMO Mo-based TMO	TMO-based hybrid material as NIB anode is highly electroactive. Nb-based transition metal oxiede(TMO) has high chemical stability Fe-based TMO exhibit high theoretical capacity, are non-toxic Co-based TMO exhibit high theoretical capacity and small volume expansion during charging-discharging Ni-based TMO has high specific capacity Cu-based TMO exhibits stable capacity. Mo-based TMO shows high cyclability TMOs hybridized with carbonaceous materials have the high specific capacity over long cycles		Low electrical conductivity, poor ion diffusivity (TMOs-carbon) Concomitant severe pulverization phenomenon (Fe-based TMO) Low electrical conductivity, poor cycling stability (Co-based TMO) Sluggish kinetics (Ni-based TMO)	Anode	NIB	[22]
TMSs-carbon Mo-based TMSs Fe-based TMSs Co-based TMSs Ni-based TMSs Sn-based TMSs	TMSs-carbon hybrid structures for NIB anode. Despite the recent significant progress made in the synthesis of TMSs		There is still a lot of areas that could be explored for achieving better results for NIB negative electrode.	Anode	NIB	[22]
Phosphorene– graphene	A sandwiched phosphorene–graphene hybrid material as a high-capacity anode for NIB shows an 83% capacity retention. The presence of graphene layers in the hybrid material works as a mechanical backbone and an electrical highway	2440 mAh g^−1^ at a current density of 0.05 A g^−1^ after 100 cycles	Relatively low first-cycle coulombic efficiency of 80%	Anode	NIB	[23]
TiNb_2_O_7_- graphene	TiNb_2_O_7_-graphene (TNO-TG) hybrid nanomaterial as an anode for LIBs with high rate capability (Coulombic efficiency of 80% at 16 C), high safety	230 mAh g^−1^ after 50 cycles at 0.1 C	Relatively high resistances	Anode	LIB	[24]
LiFePO_4_(LFP)- graphite	LFP/graphite-20% cathode electrode delivered 51.0% of the capacity retention, and the capacity returned back to its initial value when the current density was reduced to 1 C, suggesting the excellent reversibility of both Li^+^ and PF_6_^-^ storage in the hybrid material.	78.7 mAh g^−1^ at 20 C	Low capacity retention	Cathode	LIB	[25]

**Table 2:** Polymeric nanocomposites (PNCs) systems applied in Lithium-ion batteries (LIBs) and supercapacitors as electrode materials.

Electrochemical System	Description of PNC		Reference
	Lithium-ion cell	Specific capacity (mAh g^−1^)	
	Cathode		
V_2_O_5_/PPy	The PPy layer on the surface of V_2_O_5_ plays a role of plastic protecting shell, and the collapse of V_2_O_5_ due to volume expansion during the charge/discharge process can be prevented		[29]
UGF(ultrathin graphite foam)-V_2_O_5_/PEDOT core-shell	A coating of PEDOT(poly(3,4-ethylenedioxythiophene) thin shell is the key to the high performance. An excellent high-rate capability and ultrastable cycling up to 1000 cycles are demonstrated	297 at 1 C.	[30]
PTCDA/CNT	PTCDA/CNT exhibited an enhanced rate capability. Polymerization increased the cycling stability of organic cathode materials	115 at 2 C	[31]
	Anode		
rGO/SnO_2_/PANI	rGO/SnO2/PANI composite accommodate for the volume expansion during the insertion/extraction	397 at 10 A g^−1^	[32]
PANI/TiO_2_	In case of PANI/TiO_2_ coating polymer helps the particles to remain electronically connected and also creates an electrically conductive route for the electrons transfer	281 at 20 mA g^−1^	[33]
PANI/Si	In n-Si/PANI polymer can accommodate volume changes (buffers stress structure) increase the electric conductivity	561 at 0.1 C	[34]
	Supercapacitor	Specific capacitance (F g^−1^)	
PANI@ACNT(aligned small carbon nanotube)	PANI@AACNT showed high specific energy of 18.9 Wh kg^−1^ high maximum specific power of 11.3 kW kg^−1^ in an aqueous electrolyte at 1.0 A g^−1^, excellent rate performance and cycling stability	163 (only 50 for pristine CNT)	[35]
PPy/CNT	The open network of CNT-polypyrrole favors the formation of 3D double layer		[36]
PPy/RGO	The RGO provide large accessible surface area for charge separation at the electrode/electrolyte interface and PPy contribute pseudocapacitance to the energy storage	424	[37]
PANI-coated honeycomb-like MnO_2_ nanosphere	The nanocomposite cathode has a Coulombic efficiency of 77% after 1000 cycles at 8 A g^−1^	565 at 0.8 A g^−1^	[38]

**Table 3:** Electrochemical mechanism of intercalation of various graphene-based nanocomposites for LIBs, based on [42].

Nanocomposite System	Intercalation-Deintercalation Mechanism	Description
Graphene-supported transitional metal oxides	$M_{x}O_{y}+2ye^{-}+2yLi^{+}↔x{\left[M\right]}^{0}+yLi_{2}O$ When M is a transitional metal such as Ni, Co, Cu, Fe or Mn, the final product would be a homogeneous distribution of metal nanoparticles embedded in a Li_2_O matrix.	General
Graphene–Sn/Si/Ge-based nanocomposites	$M_{x}O_{y}+2ye^{-}+2yLi^{+}↔x{\left[M\right]}^{0}+yLi_{2}O$ $M+zLi^{+}+ze^{-}↔Li_{z}M$	General
Graphene-supported metal sulfides	$MoS_{2}+xe^{-}+xLi^{+}↔Li_{x}MoS_{2}$ $\left(4-x\right)Li^{+}+Li_{x}MoS_{2}+\left(4-x\right)e^{-}↔Mo+2Li_{2}S$	Molybdenum sulfide
Graphene-supported metal sulfides	$SnS_{2}+4Li^{+}+4e^{-}↔Sn+2Li_{2}M$ $Sn+xLi^{+}+xe^{-}↔Li_{x}Sn$	Tin sulfide
Graphene-supported metal sulfides	$M_{x}S_{y}+ne^{-}+nLi^{+}↔Li_{n}M_{x}S_{y}$ $\left(2y-n\right)Li^{+}+Li_{n}M_{x}S_{y}+\left(2y-n\right)e^{-}↔x{\left[M\right]}^{0}+yLi_{2}S$ $M_{x}S_{y}+2ye^{-}+2yLi^{+}↔x{\left[M\right]}^{0}+yLi_{2}S$	Cobalt/nickel sulfide

**Table 4:** Application of nanocomposites in sodium-ion batteries (NIBs) and their electrochemical performance as electrodes, based on review [87].

Type	Nanocomposite	Specific Capacity (mAh g^−1^)	Coulombic Efficiency (%)	Reference
	Anodes			
Carbon-based	PCNF@SnO_2_@C	374 mAh g^−1^ after 100 cycles	98.9%	[88]
	CuVOH-NWs	287.4 mAh g^−1^ after 50 cycles at a current density of 0.5 A g^−1^	90%	[89]
	MnFe_2_O_4_ (MFO)@C	305 mAh g^−1^ at 10 A g^−1^ after 4200 cycles		[90]
	Bi_2_Se_3_/C	527 mAh g^−1^ at 0.1 A g^−1^ over 100 cycles	89%	[91]
	Robust Polyhedral CoTe_2_–C	323 mAh g^–1^ stable capacity retentions over 200 cycles, and fast C-rate behavior (240 mAh g^–1^ at 2 C rate)		[92]
Graphene-based	Bi@graphene	561 mAh g^−1^ at the current density of 40 mA g^−1^		[93]
	Sb/rGO	500 mAh g^−1^ at density current of 1 A g^−1^ after 100 cycles		[94]
Sulfide-based	MoS_2_/SnS_2_	750 mAh g^−1^ and 600 mAh g^−1^ after 100 cycles at the current density of of 0.1 A g^−1^	89%	[86]
	MoS_2_/PEO	225 mAh g^−1^ under a current density of 50 mA g^−1^, twice as high as that of commercial MoS_2_ (com-MoS_2_), improved rate performance due to enhanced Na-ion diffusivity	90%	[85]
	SnS/C	400 mAh g^−1^ at 800 mA g^−1^		[94]
Black phosphorus-based	Black phosphorus (BP)/Ti_3_C_2_ MXene	774.4 mAh g^−1^ was achieved in the 2^nd^ cycle at a current density of 0.1 A g^−1^		[95]
Cobalt-based	Dual-meso Co_3_O_4_	267–416 mAh g^−1^ at (2430–90 mA g^−1^ respectively) after 100 cycles		[96]
	Cathodes			
Metal oxides	Na_0.33_V_2_O_5_ nanosheet@graphene	213 mAh g^−1^ at 20 mA g^−1^, good cycling stability, at 50 mA g^−1^ after 100 cycles	83.3%	[97]
Polyanionic compounds	NVP@rGO	118 mAh g^−1^ at 0.5 C, superior rate capability of 73 mAh g^−1^ at 100 C	70.0%	[98]
	RuO_2_-coated Na_3_V_2_O_2_(PO_4_)_2_F	120 mAh g^−1^ at 1 C and 95 mAh g^−1^ at 20 C after 1000 cycles		[99]
	Na_2_FeP_2_O_7_-CNTs	86 mAh g^−1^ after 140 cycles at 1 C and 68 mAh g^−1^ at 10 C		[100]
Vanadium-based polyanionic compounds	Na_3_V_2_O_2_(PO_4_)_3_/C-Ag	114.9 mAh g^−1^ at 0.2 C		[101]

**Table 5:** Various application of nanocomposites in fuel cells.

Electrode System	Application	Fuel Cell Performance (W cm^−2^)	Reference
ZnO-NiO	Low-temperature solid oxide fuel cells (LTSOFC)	1107	[162]
Two-chamber (microbial fuel cells) MFC N-doped graphene/CoNi alloy within bamboo-like CNT hybrid	MFC	2000	[163]
LSM–YSZ	Highly durable solid oxide fuel cell (SOFC) cathodes	0.65 0.55	[164]
Cu_0.15_Ni_0.85_-GDC (gadolinium doped cerium)	LTSOFC	0.82	[165]
Metal oxides Ni–Cu–Zn-oxide and samarium doped ceria-carbonate nanocomposite	LTSOFC, 300–600 °C	0.73	[166]
Hierarchically structured textile polypyrrole/poly(vinyl alcohol-co-polyethylene)nanofibers/poly(ethylene terephthalate)	Two-chamber MFC	0.42	[167]
Tailored unique mesopores, carbon nanofiber aerogel	Two-chamber MFC	0.18	[168]
Chitosan-dispersed multiwalled carbon nanotubes	Two-chamber MFC	0.29	[169]
PANI/reduced graphene oxide (rGO)/Pt	Two-chamber MFC	0.21	[170]
N-doped graphene/CoNi alloy within bamboo-likeCNT hybrid	Two-chamber MFC	0.20	[171]
N-Ni-Carbon nanofiber (CNF)/activated carbon fiber	Two-chamber MFC	0.19	[172]
N-Ni-CNF coated with poly(dimethylsiloxane)	Single-chamber MFC	0.17	[173]

**Table 6:** Photovoltaic nanocomposites as electrodes, basic structures, and efficiency.

Components	Basic Structure	Photovoltaic Device	PCE (%)	Reference
Dithienol [3,2-b:20,3d]pyrrole)-alt -4,7-(2,1,3-benzothiadiazole-PDTPBT:PbS_x_Se_1−x_	Nanocrystals	Hybrid solar cell	5.5	[180]
PCPDTBT:CdSe	Nanorods	Hybrid photovoltaic cell	5.2; 4.7	[181]
PPV:CdTe	Nanocrystals	Aqueous-solution-processed hybrid solar cell	4.76	[182]
P_3_HT:CdS	Quantum dots: QDs + nanowire of P_3_HT	Inorganic-organic hybrid solar cell	4.1	[183]
Fluorine tin oxide-FTO/PEDOT:PSS/P_3_HT:PCBM/TiO_2_	Nanotube array of TiO_2_	Double heterojunction solar cell	4.18	[184]
FTO/PEDOT:PSS/P_3_HT:S_Q-1_/TiO_2_	Nanotube array of TiO_2_	Heterojunction solar cell	3.8	[185]
MoS_2_/graphene	Uniform spherical shaped nanoparticles	Dye-sensitized solar cell	8.92	[186]
TiO_2_-2%G	Nanocomposite	Dye-sensitized solar cell	7.68	[187]
G-ZnO	Graphene layer and ZnO nanosheets	Dye-sensitized solar cell	7.01	[188]
PRGO-PTB7-th (thieno[3,4-b]thiophene.benzodithiophene)	Covalently aliphatic polymer-grafted reduced graphene oxide hybrids	Inorganic-organic hybrid solar cell	7.24	[189]
TiO_2_/silver/carbon nanotube	Nanocomposite with Ag nanoparticles	Dye-sensitized solar cell	3.76	[190]

## Data Availability

All drawings were made by myself!
